# Microsatellite Length Scoring by Single Molecule Real Time Sequencing – Effects of Sequence Structure and PCR Regime

**DOI:** 10.1371/journal.pone.0159232

**Published:** 2016-07-14

**Authors:** Mikkel Meyn Liljegren, Eric Jacques de Muinck, Pål Trosvik

**Affiliations:** Centre for Ecological and Evolutionary Synthesis, Dept. of Biosciences, University of Oslo, Oslo, Norway; University of Helsinki, FINLAND

## Abstract

Microsatellites are DNA sequences consisting of repeated, short (1–6 bp) sequence motifs that are highly mutable by enzymatic slippage during replication. Due to their high intrinsic variability, microsatellites have important applications in population genetics, forensics, genome mapping, as well as cancer diagnostics and prognosis. The current analytical standard for microsatellites is based on length scoring by high precision electrophoresis, but due to increasing efficiency next-generation sequencing techniques may provide a viable alternative. Here, we evaluated single molecule real time (SMRT) sequencing, implemented in the PacBio series of sequencing apparatuses, as a means of microsatellite length scoring. To this end we carried out multiplexed SMRT sequencing of plasmid-carried artificial microsatellites of varying structure under different pre-sequencing PCR regimes. For each repeat structure, reads corresponding to the target length dominated. We found that pre-sequencing amplification had large effects on scoring accuracy and error distribution relative to controls, but that the effects of the number of amplification cycles were generally weak. In line with expectations enzymatic slippage decreased proportionally with microsatellite repeat unit length and increased with repetition number. Finally, we determined directional mutation trends, showing that PCR and SMRT sequencing introduced consistent but opposing error patterns in contraction and expansion of the microsatellites on the repeat motif and single nucleotide level.

## Introduction

Microsatellite DNA, also known as short tandem repeats (STR) or simple sequence repeats, are tracts of mono- to hexanucleotide repeats of variable length that are widely distributed in the genomes of animals, plants and microbes [1]. Microsatellites are usually found in non-coding genomic regions and are generally thought to be evolutionarily neutral, although they can have large phenotypic effects in specific scenarios [2]. Microsatellite evolution is commonly described by a stepwise mutation model [3], in which transient dissociation of two replicating DNA strands leads to looping by one or more repeat units [4]. The looping of one strand, with subsequent misaligned re-association, leads to enzymatic slippage resulting in microsatellite expansion if looping occurs on the nascent strand and contraction if it occurs on the template strand. Although many details concerning the microsatellite mutation process are still unclear, there is experimental evidence that DNA polymerase activity is the main mechanism [5,6].

Due to their high degree of allelic variability, microsatellites are popular markers in population genetics [7], linkage mapping [8], forensic analysis [9], cancer diagnostics and prognosis [10] and strain level typing of bacteria [11]. Another use of microsatellite sequences is as plasmid-carried indicators of adaptation rates in bacterial evolution experiments [12,13]. Microsatellite lengths are typically measured by PCR amplification followed by some form of high resolution electrophoresis, amplicon visualization and size determination against a known standard. Currently, the fastest and most accurate method is PCR with a fluorescently labelled primer followed by high-voltage capillary electrophoresis and automated CCD (charge-coupled device) camera fluorescence detection [14]. Considering the decreasing costs of next-generation sequencing (NGS), as well as increasing accessibility, these technologies could represent a viable alternative to electrophoresis-based techniques [15,16,17].

SMRT sequencing [18], as implemented in the PacBio series of sequencing apparatuses, utilizes zero-mode waveguides, nanostructures with an aperture small relative to the wavelength of light, with a single DNA polymerase fixed to the bottom of each structure, enabling the observation of a single nucleotide being incorporated into the growing strand. The DNA template/insert is circularized using the SMRTbell library principle [19]. This molecule is then passed through the enzyme multiple times, producing a circular consensus sequence (CCS). The quality of the CCS depends on the number of passes, which is inversely proportional to the length of the insert.

Here, we evaluate the potential of PacBio SMRT sequencing for highly multiplexed microsatellite length scoring. This technology offers moderate output relative to other NGS platforms, but at a considerably lower cost, making it appropriate for projects where the number of required sequence reads run only into the tens of thousands, but that may require longer reads that that produced by e.g. the Illumina platform. Essentially a microsatellite length scoring protocol using SMRT sequencing would be analogous to regular amplicon sequencing. The marker of interest is amplified by PCR using primers containing an additional short index sequence specific for the sample being amplified. PCR products are then quantified before pooling all of the samples, each containing a unique index sequence. The pool is cleaned before use as input for SMRTbell library construction per the manufacturer’s instruction, followed by the actual sequencing step. The output sub-reads are then subject to further analyses in order to generate CCS and carry out quality filtering. The resulting sequence file is demultiplexed using the list of unique sample indices, before length scoring and other biological analysis.

When performing typical microsatellite length scoring analysis, artificially introduced polymorphisms are commonly introduced as a result of PCR amplification prior to scoring [1]. The rates at which these errors occur are expected to be in proportion to the PCR cycle number as additional cycles entail more interaction between DNA polymerase and template, and thus a higher potential for enzymatic slippage. Furthermore, *In vitro* experiments with *Taq* polymerase have shown that slippage rates increase with allele length while decreasing with repeat unit length, and that slippage is biased toward microsatellite contraction [20]. Here, we investigate the effects of PCR steps prior to sequencing, as well as the effects of microsatellite structure, on scoring accuracy. Finally, we examine the directionality of indel mutations in microsatellite sequences and compare this with indel patterns observed in non-microsatellite control sequences.

## Material and Methods

### Construct design

We designed two DNA constructs, each containing two microsatellites. Construct 1 (S1A Fig) is composed of a 40 bp tetranucleotide (10xATCT) and an 80 bp tetranucleotide (20xATCT) separated by a 140 bp unique linker sequence, and flanked by PCR priming sites and PstI restriction sites. Construct 2 (S1B Fig) is composed of a 40 bp tetranucleotide (10xATCT) and a 60 bp dinucleotide (30xCA) separated by a 133 bp unique linker sequence, and flanked by PCR priming sites and EcoRI restriction sites. These three microsatellite sequences were chosen in order to represent different mutation rates. Constructs were ordered from GenScript and delivered as lyophilized DNA inserted in plasmid backbone pUC57 [21]. Quality control and sequencing of constructs were performed by GenScript.

### Cloning and transformation

Constructs were excised from pUC57 by EcoRI or PstI digestion (NEB, digestions done in 30 μl volumes consisting of 5 μl 0.2 μg/μl DNA, 3 μl 10x NEB 3 buffer, 1 μl (20,000 U/ml) PstI or EcoRI enzyme and 21 μl H_2_O, incubation at 37°C for 4 hours). Digested fragments were separated by electrophoresis on a 1% agarose gel. Constructs were isolated using the QIAquick gel extraction kit according to instructions supplied by the manufacturer. Plasmid pBR329 [22] (S2A Fig) was digested using either EcoRI or PstI as described above, followed by shrimp alkaline phosphatase (SAP) treatment (Fermentas, 40 μl vector, 4.6 μl 10x SAP buffer, 1.4 μl SAP 1 U/μl, 60 min. at 37°C). Plasmids and constructs were ligated (NEB, 3 μl 13 ng/μl pBR329, 2 μl 5 ng/μl construct, 2 μl T4 ligase buffer, 0.8 μl T4 ligase (400,000 U/ml), 12 μl mqH_2_O), producing plasmids pBR329-C1 (with construct 1) and pBR329-C2 (with construct 2) carrying disrupted resistance markers for ampicillin and chloramphenicol, respectively (S2B and S2C Fig). Cloned construct sequences were verified by Sanger sequencing (GATC Biotech, Constance, Germany). pBR329-C1 and pBR329-C2 were transformed into *E*. *coli* strain MG1655 by use of the Inoue protocol for plasmid transformation [23]. Transformants were isolated on 30 μg/ml chloramphenicol (pBR329-C1) or 50 μg/ml ampicillin (pBR329-C2) Luria Bertani (LB) plates.

### Validation of transformants

Transformation was verified in two ways. First, positive isolates were grown overnight in 10 μg/ml tetracycline LB medium at 37°C followed by plasmid extraction using the QIAGEN Plasmid Mini Kit according to instructions provided by the manufacturer. Plasmids were digested with either PstI (pBR329-C1) or EcoRI (pBR329-C2) as described above, using 5 μl of plasmid extract. Presence of the inserts was evaluated by visualization of appropriate size bands on a 1% agarose electrophoresis gel. Second, insert presence was verified by dissolving positive colonies in 25 μl of mqH_2_O and using this as template for PCR amplification with primer sets targeting construct flanks (S1 Table) (1 μl template, 1.5 μl 10x HF buffer, 0.2 μM forward primer, 0.2 μM reverse primer, 40 μM dNTP, 0.2 μl 2 U/μl Thermo Fisher DynaZyme II polymerase, 11.4 μl mqH_2_O, at 95°C for 7 min., 35 cycles of 95°C for 30 sec., 58°C for 30 sec. and 72°C for 1 min., final extension at 72°C for 5 min.). PCR product was evaluated by 1% agarose gel electrophoresis. Successful MG1655 transformants with either pBR329-C1 or pBR329-C2 were stored at -80°C in 20% glycerol LB. These frozen cell stocks were used for all subsequent cultures for plasmid extractions.

### Polymerase chain reaction regimes

Frozen culture stocks were grown overnight in selective 37°C LB medium (10 μg/ml tetracycline + 100 μg/ml ampicillin for pBR329-C1, or 25 μg/ml chloramphenicol for pBR329-C2) followed by extraction of pBR329-C1 and pBR329-C2 using the QIAGEN Plasmid Midi Kit according to the manufacturer's manual. Plasmid extractions were confirmed by 1% agarose gel electrophoresis and quantified using a NanoDrop ND-1000 spectrophotometer (v. 3.3). PCR was conducted using primer pairs specific for the constructs’ flanks (S1 Fig, S1 Table) in 50 μl reactions (100ng template (approx. 2e10 molecules per reaction), 10 μl 5x HF buffer, 0.5 μM each forward and reverse primers, 40 μM dNTP, 2 mM MgCl_2_, 2 μl DMSO, 0.5 μl 2 U/μl Thermo Scientific Phusion High-Fidelity polymerase). Forward primers were appended 8 bp barcodes at the 5' end to allow for multiplexed sequencing (S2 Table). Reactions were run with the following program: Initial denaturation at 98°C for 1 min., 98°C for 1 min., 56°C for 1 min. and 72°C for 1 min., final extension at 72°C for 10 min. PCR was run using 10, 20, 30 and 40 cycles of amplification with fewer-cycle PCRs carried out in replicates in order to compensate for decreased yield, totaling 200 μl for 10 cycles (four 50 μl reactions, pooled), 150 μl for 20 cycles (three 50 μl reactions, pooled), 100 μl for 30 cycles (two 50 μl reactions, pooled) and 50 μl for 40 cycles (one reaction). All of the above PCR regimes were carried out in duplicate for a total of 16 (two for each of the four PCR regimes for both constructs). PCR products were confirmed by 1% agarose gel electrophoresis.

In order to determine if the PCR reactions were saturated at high cycle numbers we ran three additional reactions for each cycle regime for each construct, using the same DNA stocks as for the sequencing experiment. Yields were then quantified with a Life Technologies Qbit fluorometer (model 1.0).

### Preparation of no-PCR control

In order to distinguish between errors introduced by SMRT sequencing and PCR, no-PCR controls were created by excising constructs from pBR329-C1 and pBR329-C2 by PstI and EcoRI enzyme digestion, respectively. Plasmid-carrying strains were grown overnight from the same frozen stocks at 37°C in 50 ml selective LB media (100 μg/ml Ampicillin + 10 μg/ml Tetracycline or 10 μg/ml Chloramphenicol and 10 μg/ml Tetracycline), i.e. the exact same conditions as above. Plasmids were extracted from cultures using the QIAGEN Plasmid Midi Kit and re-suspended in 50 μl mqH_2_O. Plasmid purification was confirmed by running 5 μl of DNA on a 1% agarose gel. Constructs were excised from plasmids by EcoRI and PstI (20,000 U/ml) digestion enzymes by adding 5 μl x10 NEB3 buffer and 0.5 μl of appropriate enzyme and digesting overnight at 37°C. Plasmid backbones and inserts were separated on a 1% agarose gel, and fragments were visualized using a Safe Imager Transilluminator (Invitrogen). Inserts were subsequently excised and purified using a QIAquick gel extraction kit.

### PacBio sequencing and data analysis

The four PCR reactions from each of the four cycle number regimes were pooled and cleaned using a QIAquick PCR Purification Kit. Each pool was quantified using a Life Technologies Qbit fluorometer (model 1.0) according to standard manufacturer's protocol and diluted with mqH_2_O to equal concentrations. The four pools were then combined in equal volumes for a total of 222 ng PCR amplicons and 13.9 ng of each no-PCR control for a total of 249.8 ng of DNA. The resulting pool was purified using the Pacific Biosciences AMPure PB prep kit according to instructions supplied by the manufacturer, and the final SMRT sequencing library was prepared using the Pacific Biosciences 500bp library preparation protocol. Fragment size was confirmed on an Agilent 2100 Bioanalyzer, and the library was sequenced on a PacBio RS II instrument using P4-C2 chemistry on one SMRT cell, with a movie time of 180 minutes. Quality filtering was performed using the “Reads Of Insert” protocol on SMRT portal (SMRTAnalysis version v2.2.0.p1 build 134282). Filtering was done with the minimum number of passes set to 3 for circular consensus sequences (CCS) (S3 Table) and a minimum predicted accuracy of 0.9. This resulted in 39,213 reads with a mean read length of 304bp, a mean read quality (QV) of 0.9952, and a mean number of passes of 26.11. The raw sequence data can be downloaded as S1 File. Sequence data were then imported into R [24] and sequences outside the 300–400 bp range were discarded. This filtering step further reduced the number of reads to 28,762, mainly due to a large number of sequence stubs that passed initial quality filtering. De-multiplexing (allowing no index mismatches) and sequence length variant calling were carried out using custom R scripts (S2–S4 Files). Since the no-PCR controls did not have dedicated barcodes for de-multiplexing, the first 8 bp after their respective restriction cut sites were used. Microsatellite length scoring was done by first trimming flanking priming sites and then counting the number of nucleotides in the repeat regions. The mean number of sequence reads per sample was 1484±1252 (SD) reads (S3 Fig). All statistical treatment comparisons were carried out using the paired or unpaired Wilcoxon signed rank sum test or generalized additive models (GAMs). GAMs were computed using the “mgcv” package for R [25], assuming a normal error distribution and using 3 degrees of freedom for smooth term estimation.

## Results

### Overall distribution of stepwise slippage errors

**Fig 1:**
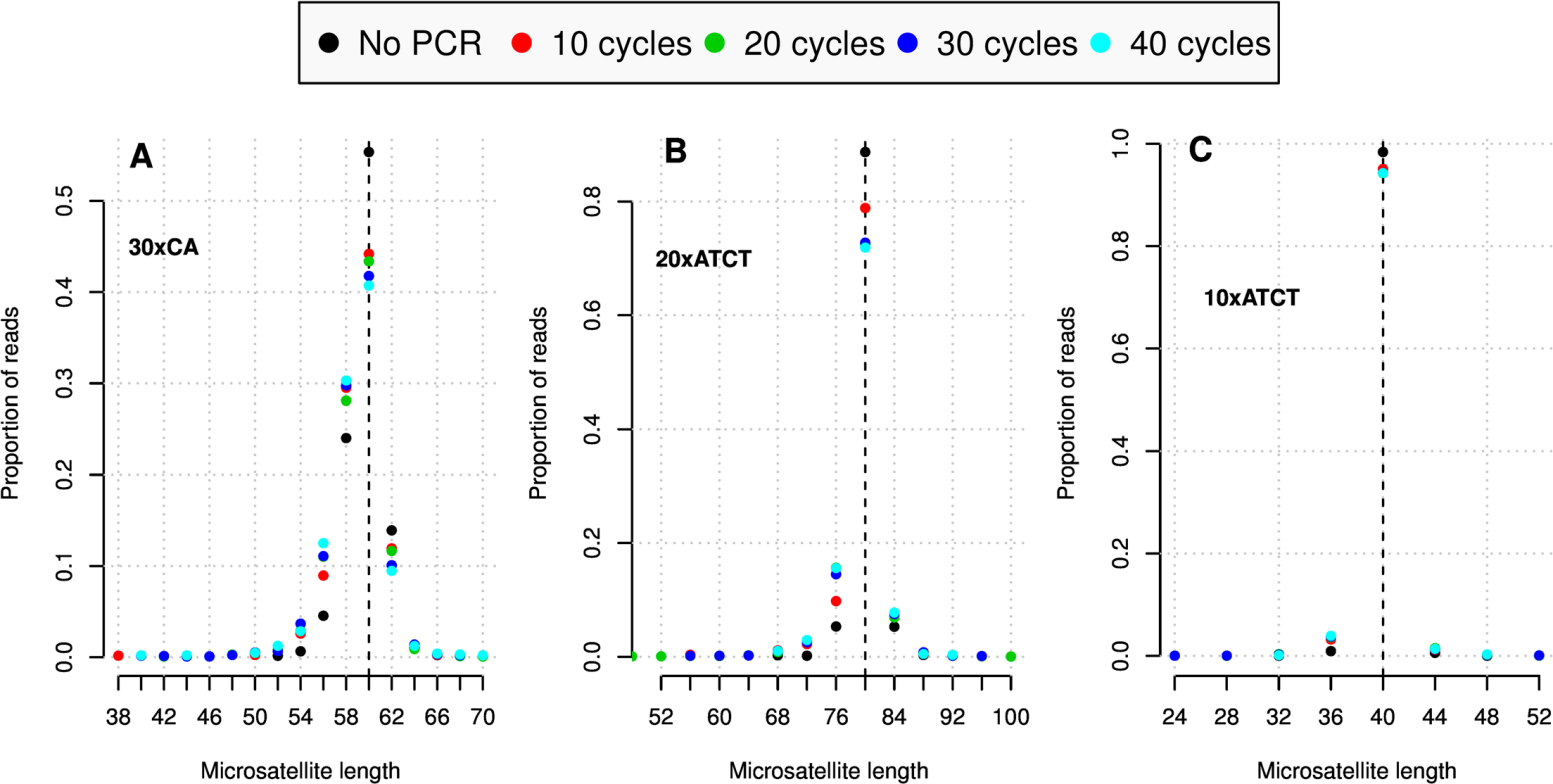
Mean distribution of enzyme slippage errors. Each dot in the graphs is a proportion of reads (y-axes) corresponding to the microsatellite lengths (bp) indicated on the x-axes. The dots are color coded according to the number of PCR cycles indicated above. A. Mean of two replicates of 30xCA repeat sequence. B. Mean of two replicates of 20xATCT repeat sequence. C. Mean of four replicates of 10xATCT repeat sequence. Singleton reads and reads with lengths not corresponding to multiples of the repeat units are not shown.

**Fig 2:**
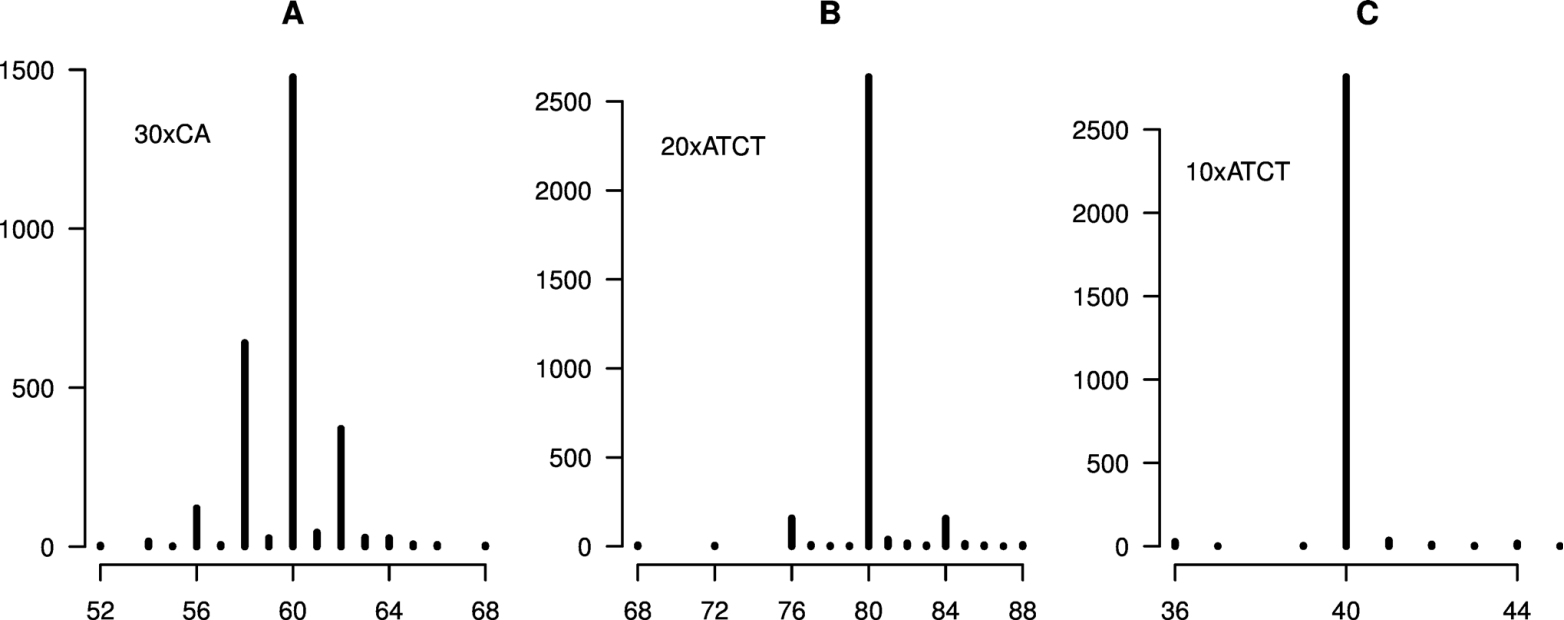
Microsatellite length distributions for no-PCR controls. A. 30xCA. B. 20xATCT. C. 10xATCT (mean of scores from constructs 1 and 2). The y-axes indicate the number of SMRT sequence reads corresponding to the sequence length (bp) indicated on the x-axes. Singleton read lengths have been omitted from the plots for enhanced visualization (see S4, S9, S14 and S19 Figs for full details).

**Fig 3:**
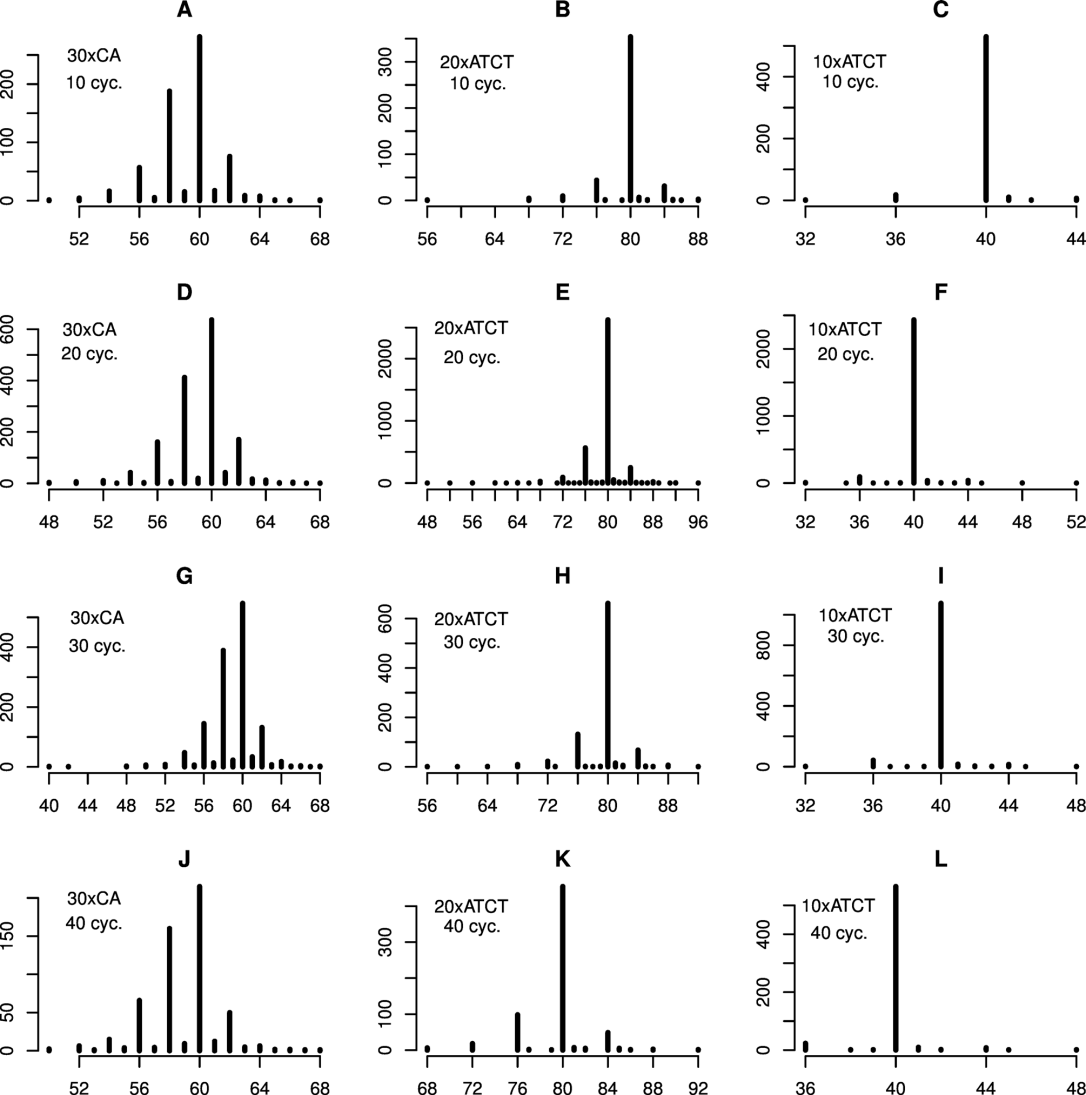
Mean microsatellite length distributions for PCR amplified fragments. A, D, G and J: 30xCA. B, E, H and K: 20xATCT. C, F, I and L: 10xATCT. A-C: 10 PCR cycles. D-F: 20 PCR cycles. G-I: 30 PCR cycles. J-L: 40 PCR cycles. All panels show mean results from two replicate PCR reactions. The y-axes indicate the number of SMRT sequence reads corresponding to the sequence length (bp) indicated on the x-axes. In each panel the target sequence length can be seen to dominate. Singleton read lengths have been omitted from the plots for enhanced visualization (see S5–S8, S10–S13, S15–S18 and S20–S23 Figs for full details).

The average distributions of slippage errors by integer numbers of repeat units, for all three microsatellite structures, are presented in Fig 1. Full distributions of read lengths for all replicates are presented in S4–S23 Figs. This information is also summarized in Figs 2 and 3. DNA polymerase slippage was more pronounced in the dinucleotide repeat sequence than the tetranucleotide, and more mutations were observed in the longer tetranuclotide sequence than the shorter. Errors leading to sequence contraction were more common than those leading to expansion. Furthermore, there was a clear tendency for fewer errors to occur in the no-PCR control samples, indicating a negative effect of pre-sequencing PCR amplification. In order to test if the combination of repeat sequences within the same construct may have influenced microsatellite stability, we compared slippage patterns between the 10xATCT sequences present on both constructs. Unpaired Wilcoxon signed rank tests comparing absolute errors (absolute differences between observed and target read length) showed no significant differences in error rates between the 10xATCT construct paired with either the 20xATCT microsatellite or the 30xCA microsatellite.

### Effect of PCR on total error patterns

**Fig 4:**
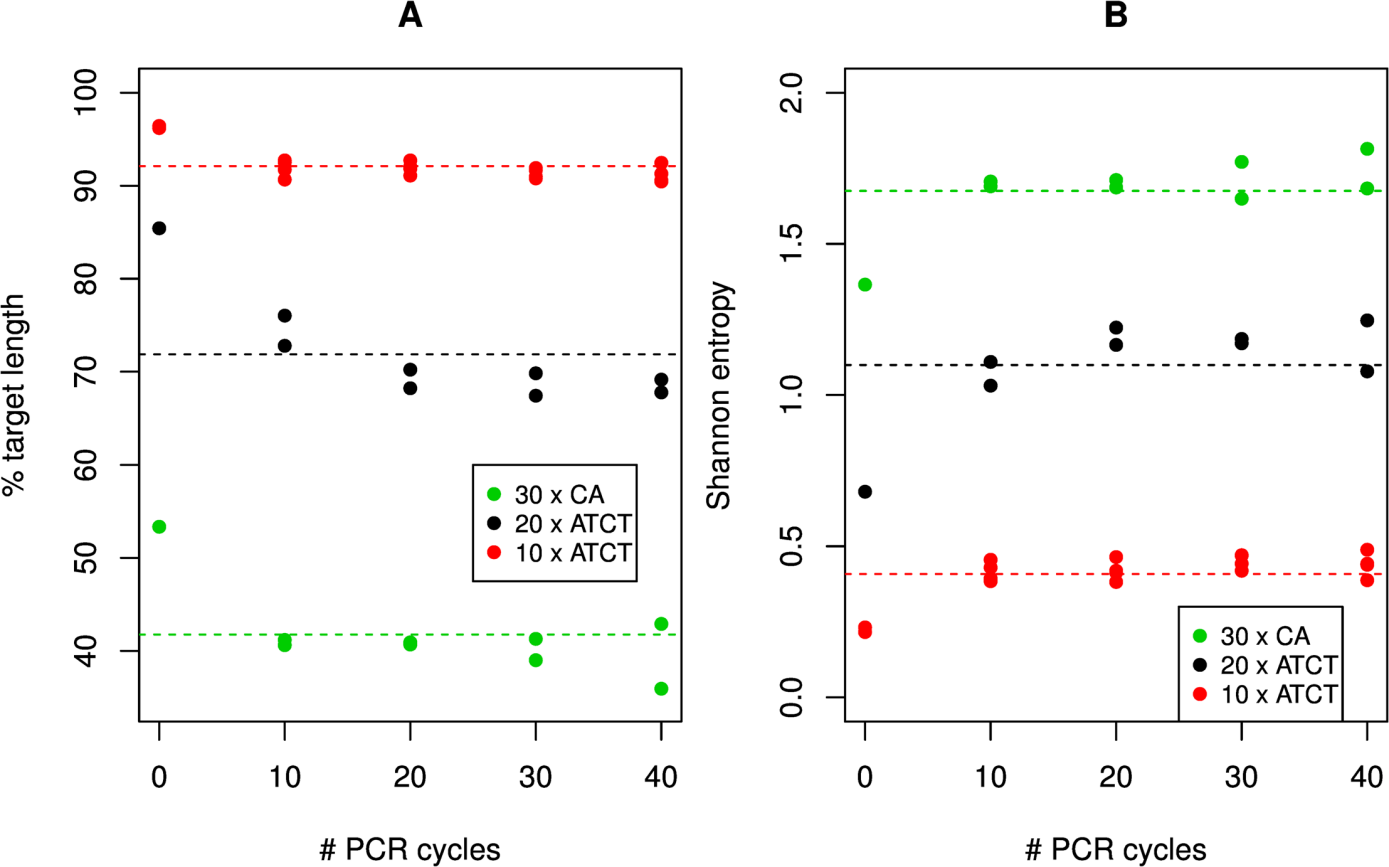
Microsatellite length scoring accuracy. A. Percentage of reads matching the expected sequence length. B. Shannon entropy of measured microsatellite lengths in each experiment. The plotted values are based on both stepwise and non-stepwise errors.

We first assessed the effects of PCR relative to the no-PCR controls by comparing the absolute errors of control reads to reads resulting from different PCR regimes (S24 Fig), including both stepwise slippage errors and errors resulting in read lengths not corresponding with an integer number of repeat units (i.e. 2 or 4). In each comparison we found that pre-sequencing PCR increased errors in a highly significant way (p<<0.001 for all comparisons, unpaired Wilcoxon signed rank test). Since the absolute error is highly dependent on microsatellite repeat unit length, we assessed microsatellite length scoring accuracy in two more ways for easier comparison between microsatellite structures. First we looked at the percentage of sequence reads that matched the original length (Fig 4A). The restriction digest controls form high leverage points when modelling this as a function of the number of PCR cycles, leading to significant or marginally significant models (S25A–S25C Fig) (30xCA: p = 0.058, 20xATCT: p<0.001, 10xATCT: p<0.001; GAM). After removing the leverage points, two of the models were no longer significant (30xCA: p = 0.479, 10xATCT: p = 0.123), while one was marginally significant (20xATCT: p = 0.028) (S25D–S25F Fig). As an alternative measure of accuracy we used Shannon entropy. The Shannon entropy is zero if all sequence reads are of a single (target) length, and increases as the read length distribution becomes more diverse. We observed a pronounced increase in spread from the no-PCR controls to the experiments using PCR prior to sequencing (Fig 4B). Using the modelling approach described above we again saw a significant to marginally significant relationship with the number of PCR cycles (30xCA: p = 0.059, 20xATCT: p = 0.002, 10xATCT: p<0.001) (S25A–S25C Fig). When the restriction digest controls were left out this relationship was no longer observable (30xCA: p = 0.373, 20xATCT: p = 0.4, 10xATCT = 0.185) (S26D–S26F Fig), indicating that increasing the number of PCR cycles has little effect on microsatellite length scoring accuracy. Yield measurements for all PCR cycle regimes for both constructs did show indication, albeit weak, of PCR saturation after 30 cycles (S27 Fig).

### Directional trends in stepwise mutation patterns

**Fig 5:**
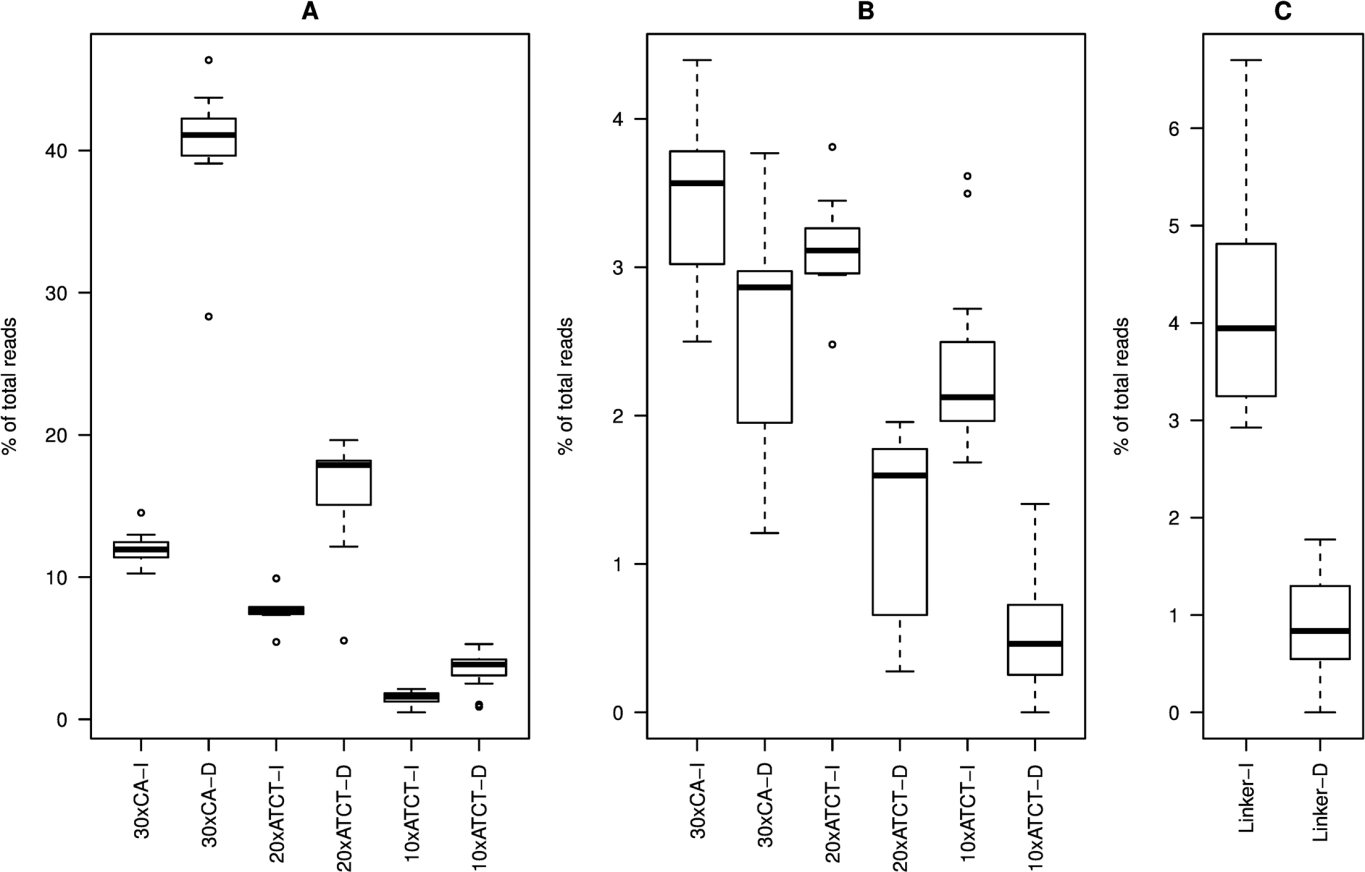
Directionality of indel mutation events. Percentages of non-target read lengths are on the y-axes, while the microsatellite structures and directionality of mutation events (I = insertion; D = deletion) are indicated on the x-axes. The boxes represent the inter-quartile range with the thick lines representing the medians. The whiskers are 1.5 times the inter-quartile range and outliers are plotted as individual dots. A. Stepwise mutations by integer numbers of repeat units. B. Non-stepwise mutations. C. Indel mutations by one or more bp in linker sequences.

For each PCR regime we calculated the percentage of non-target length sequence reads that corresponded with either deletions or insertions by integer numbers of repeat units (Fig 5A). Comparison of these proportions in the three microsatellite types consistently found that slippage errors leading to sequence contraction were much more common than those leading to expansion, with mean ratios of deletion to insertion events at 3.43, 2.00 and 2.45 for 30xCA, 20xATCT and 10xATCT, respectively (p = 0.004, p = 0.004 and p< 0.001; paired Wilcoxon signed rank test). We further examined the relationship between the stepwise errors and PCR amplification regime separately for deletions and insertions. In the case of the 30xCA sequence we observed opposing trends with increasing deletion rates and decreasing insertion rates with increasing numbers of PCR cycles (p = 0.004 and 0.005, respectively; GAM; S28A and S28D Fig). These relationships still held when the no-PCR control samples (high leverage points) were omitted from the models (p = 0.027 and 0.024, respectively; GAM, S29A and S29D Fig). In contrast, both stepwise deletion and insertion rates increased in PCR-amplified samples relative to no-PCR controls for the 20xATCT (p<0.001 and 0.024, respectively; GAM; S28B and S28E Fig) and 10xATCT sequences (p = 0.001 and 0.006, respectively; GAM; S28C and S28F Fig), but models were no longer significant when no-PCR controls were omitted (S29B, S29C, S29E and S29F Fig), further indicating minimal effects of PCR cycle number.

### Non-stepwise errors

We calculated the percentage of non-target length sequence reads that did not correspond with deletions or insertions by integer numbers of repeat units (Fig 5B). These events were rare relative to stepwise errors with ratios of stepwise to non-stepwise errors of 9.08, 5.57 and 2.42 for the 30xCA, 20xATCT and 10xATCT microsatellite, respectively (p<0.005 for all comparisons, paired Wilcoxon signed rank test). Interestingly, the non-stepwise errors showed the opposite trend from the stepwise slippage error patterns with a predominance of insertions for all three microsatellite types, with mean ratios of deletions to insertions of 0.72, 0.4 and 0.249 for 30xCA, 20xATCT and 10xATCT, respectively (p = 0.02, p = 0.004 and p<0.001; paired Wilcoxon signed rank test). This trend was found to be caused mainly by reads one nucleotide longer than the target length (S30 Fig), which occurred at mean rates of 2.3, 1.35 and 1.50 of reads for the 30xCA, 20xATCT and 10xATCT sequences, respectively. There was no discernible difference in effect between the no-PCR controls and the PCR amplification experiments or correlation between non-stepwise error rates and the number of PCR cycles (S31 Fig), indicating that these errors were introduced mainly during SMRT sequencing.

For comparison with non-microsatellite DNA we looked at indel patterns in the linker sequences separating the two microsatellites on each construct. These DNA sequences are essentially random and thus suitable for obtaining baseline indel rates introduced through PCR and SMRT sequencing. For these sequences we also observed a large predominance of insertion to deletion events (p<0.001, paired Wilcoxon signed rank test; Fig 5C), with a mean deletion to insertion ratio of 0.24. The overall mean percentage of reads with an indel mutation was 5.08, while for the no-PCR controls the percentage was 4.70, again indicating that these errors are introduced mainly during SMRT sequencing. As for the repeat sequences, the majority (2.8%) of indel events were caused by single nucleotide insertions (S30 Fig). There was a noisy but significant linear relationship between indel rate and the number of PCR cycles (p = 0.006, linear model) with indel rates increasing by 0.35% for each ten added cycles (S32 Fig). When normalized for sequence length indel rates in the linkers were comparable to non-stepwise indel rates in the microsatellites (S33 Fig), with the exception that the deletion rate in the 30xCA sequence was increased more than twofold relative to the tetranucleotide and linker sequences, indicating that this repeat pattern also effects the sequence’s propensity for non-stepwise mutations.

## Discussion

For all experimental treatments sequence reads corresponding to the target allele length dominated (Fig 1). However, we did observe significant numbers of reads that did not produce the expected microsatellite sequence lengths, in particular for samples having undergone pre-sequencing PCR. For the 30xCA dinucleotide sequence, PCR amplified samples had an average of 42.5% of reads measured at the expected 60 pb, with significant read numbers at 58 (29.3%), 56 (10.9%) and 62 (10.8%) bp. High numbers of spurious length variants are also seen in (CA)_n_ repeats analysed by electrophoretic techniques, which can complicate the analysis of these sequences [26]. The lengths of the tetranuclotide sequences were more accurately measured. For the PCR amplified 20xATCT sequence a mean of 74.0% of reads was the expected 80 bp, while we observed significant read numbers at 76 (13.9%) and 84 (7.3%) bp. The 10xATCT sequence was the expected 40 bp in 95% of reads with little difference between PCR amplified samples and no-PCR controls. It should be noted that there is a chance, albeit remote [12], that overnight growth in *E*.*coli* prior to plasmid extraction may have contributed to the error distributions of the PCR amplified samples and the no-PCR controls. The mutation rates we observed, with the highest rates occurring in the dinucleotide repeat sequence, and higher rates in the longer tetranuclotide sequence than the shorter, are in line with previous studies of variability of naturally occurring microsatellite alleles in genomes [27,28,29]. The spurious read lengths corresponding to integer numbers of repeat units are analogous to the so called ‘stutter bands’ observed when analysing microsatellites using capillary electrophoresis [1], and are to a large part artefacts introduced during PCR amplification. This is in accordance with results from Loomis *et al*. [30] who successfully used SMRT sequencing to analyse CGG trinucleotide repeats in fragile X syndrome, finding that PCR amplification significantly affected typing accuracy. The observed tendency for microsatellite length scoring error rates to remain unrelated to the number of PCR cycles is difficult to explain, although a tendency towards PCR saturation going from 30 to 40 cycles may partially account for this result (S27 Fig). The lack of observed effects may also be related to idiosyncrasies of the interaction between DNA polymerase and microsatellites, and it is possible that we mostly observe indel events taking place in early PCR cycles, while subsequent indels are partially masked by frequent back-mutations.

Existing literature provides somewhat conflicting information regarding the typical directionality of stepwise mutations in microsatellites. Shinde *et al*. [20] investigated the effects of PCR on mono (A) and dinucleotide (CA) repeat sequences cloned onto plasmids and found a preponderance of deletions with contraction mutations outnumbering expansions by a factor of 14 in the case of dinucleotides of up to 14 repeats in length. Eckert and Yan [31] used plasmid-carried di (CA and AG) and tetranuclotide (TTCC) microsatellites in isogenic *E*.*coli* strains negative and positive for post-replication mismatch repair, finding a roughly twofold predominance of expansions in the tetranucleotide sequence and a two to fourfold preponderance of contractions in the dinucleotides. Using a similar approach, but with a mammalian cell line, Twerdi *et al*. [32] found a predominance of insertion mutations in a plasmid-carried 17xCA microsatellite. A study of naturally occurring microsatellites in humans has found that mutation directionality was highly dependent on allele type, but that overall rates of expansion and contraction were similar [33]. Our observations were consistently in favour of deletion mutations leading to allele contraction. Non-stepwise mutation patterns in polynucleotide microsatellite sequences have not, to our knowledge, been explored previously but a study of mononucleotide microsatellite instability showed that rates of single nucleotide indels depend on the particular sequence [34].

In a previous study, PacBio SMRT sequencing was used for development of novel microsatellite marker sequences [35]. This approach involved a genomic shotgun approach which entails stringent demands on DNA quality. Microsatellite length scoring by SMRT sequencing of amplicons, as well as traditional fragment analysis by capillary electrophoresis, only require that the DNA is of PCR quality, making these techniques accessible for population genetic studies. Both SMRT sequencing and methods based on capillary electrophoresis face some similar technical challenges. In particular the effects of enzymatic slippage during PCR are observed in both techniques; as ‘stutter bands’ in capillary electrophoresis and as off-target read lengths in SMRT sequencing. In terms of performance, both techniques are able to correctly identify dominant fragment lengths. Current cost estimates are also comparable at roughly 300USD per 100 samples (personal communication with commercial service providers, including PacBio library preparation, but excluding costs of primers and other PCR reagents for both PacBio sequencing and capillary electrophoresis). One challenge, as observed in this study, when multiplexing large numbers of samples in a SMRT sequencing run, is combining equal amounts of input DNA per sample. Poor normalization may result in a lot of wasted reads, leading to samples with insufficient read numbers for confident variant calling. Given an effective normalization strategy, however, the technique described here is easily scalable to larger datasets. For a population genetics study, 100 sequence reads per marker for one sample should be sufficient to establish genotype. Thus, with the sequencing output achieved here, up to 300 samples could be analysed on a single SMRT cell (current PacBio technology accommodates up to 16 SMRT cells in a single run), and output is steadily increasing. Further, using dual indexing for demultiplexing both on the forward and reverse primers, 300 samples can be analysed using only 35 short oligos. Note that the same number of oligos would be required for each marker analysed, e.g. a total of 350 oligos for a typical population genetic study using 10 microsatellite markers. It should also be noted that the current PacBio P6/C4 chemistry has increased the average read length to 10,000–15,000 bp, which will increase the length of markers that can be scored with confidence. Further, the recently released PacBio Sequel System has increased the number of zero-mode waveguides from 150,000 to 1,000,000 per SMRT Cell, leading to a 7x increase in the number of output reads per run. Clearly, when analysing a small to moderate number of samples, capillary electrophoresis offers more flexibility. Other NGS platforms, such as Illumina MiSeq, may be more cost-effective for multiplexing several thousand samples. However, the throughput offered by SMRT sequencing does present this approach as a viable alternative for multiplexing hundreds of samples in a single sequencing run.

## Supporting Information

S1 Fig Schematic of constructs 1 and 2. (PNG)

S2 Fig Plasmid pBR329 and construct insertion sites. Construct 1 disrupts ampR gene, allowing for selection for ampicillin-sensitive hosts. Construct 2 disrupts camR gene, allowing for selection for chlorampenicol-sensitive hosts. Both vectors provide tetracycline resistance to host.
(PNG)

S3 Fig Number of reads in each experiment. The total number of reads for each experimental treatment in indicated on the y-axis. Experimental treatment is indicated is indicated on the x-axis. EcoRI and pstI are the no-PCR controls. Construct number (C1 or C2) is indicated first in the other x-axis labels, followed by replicate number (1 or 2), followed by number of PCR cycles (10–40).
(BMP)

S4 Fig Repeat length distribution for 30xCA sequence, EcoRI restriction digest control. The y-axis indicates the number of SMRT sequence reads corresponding to the sequence length (bp) indicated on the x-axis.
(BMP)

S5 Fig **Repeat length distribution for 30xCA sequence, 10 PCR cycles replicates 1 (A) and 2 (B).** The y-axis indicates the number of SMRT sequence reads corresponding to the sequence length (bp) indicated on the x-axis.
(BMP)

S6 Fig **Repeat length distribution for 30xCA sequence, 20 PCR cycles replicates 1 (A) and 2 (B).** The y-axis indicates the number of SMRT sequence reads corresponding to the sequence length (bp) indicated on the x-axis.
(BMP)

S7 Fig **Repeat length distribution for 30xCA sequence, 30 PCR cycles replicates 1 (A) and 2 (B).** The y-axis indicates the number of SMRT sequence reads corresponding to the sequence length (bp) indicated on the x-axis.
(BMP)

S8 Fig **Repeat length distribution for 30xCA sequence, 40 PCR cycles replicates 1 (A) and 2 (B).** The y-axis indicates the number of SMRT sequence reads corresponding to the sequence length (bp) indicated on the x-axis.
(BMP)

S9 Fig Repeat length distribution for 20xATCT sequence, PstI restriction digest control. The y-axis indicates the number of SMRT sequence reads corresponding to the sequence length (bp) indicated on the x-axis.
(BMP)

S10 Fig **Repeat length distribution for 20xATCT sequence, 10 PCR cycles replicates 1 (A) and 2 (B).** The y-axis indicates the number of SMRT sequence reads corresponding to the sequence length (bp) indicated on the x-axis.
(BMP)

S11 Fig **Repeat length distribution for 20xATCT sequence, 20 PCR cycles replicates 1 (A) and 2 (B).** The y-axis indicates the number of SMRT sequence reads corresponding to the sequence length (bp) indicated on the x-axis.
(BMP)

S12 Fig **Repeat length distribution for 20xATCT sequence, 30 PCR cycles replicates 1 (A) and 2 (B).** The y-axis indicates the number of SMRT sequence reads corresponding to the sequence length (bp) indicated on the x-axis.
(BMP)

S13 Fig **Repeat length distribution for 20xATCT sequence, 40 PCR cycles replicates 1 (A) and 2 (B).** The y-axis indicates the number of SMRT sequence reads corresponding to the sequence length (bp) indicated on the x-axis.
(BMP)

S14 Fig Repeat length distribution for 10xATCT sequence in construct 1, PstI restriction digest control. The y-axis indicates the number of SMRT sequence reads corresponding to the sequence length (bp) indicated on the x-axis.
(BMP)

S15 Fig **Repeat length distribution for 10xATCT sequence in construct 1, 10 PCR cycles replicates 1 (A) and 2 (B).** The y-axis indicates the number of SMRT sequence reads corresponding to the sequence length (bp) indicated on the x-axis.
(BMP)

S16 Fig **Repeat length distribution for 10xATCT sequence in construct 1, 20 PCR cycles replicates 1 (A) and 2 (B).** The y-axis indicates the number of SMRT sequence reads corresponding to the sequence length (bp) indicated on the x-axis.
(BMP)

S17 Fig **Repeat length distribution for 10xATCT sequence in construct 1, 30 PCR cycles replicates 1 (A) and 2 (B).** The y-axis indicates the number of SMRT sequence reads corresponding to the sequence length (bp) indicated on the x-axis.
(BMP)

S18 Fig **Repeat length distribution for 10xATCT sequence in construct 1, 40 PCR cycles replicates 1 (A) and 2 (B).** The y-axis indicates the number of SMRT sequence reads corresponding to the sequence length (bp) indicated on the x-axis.
(BMP)

S19 Fig Repeat length distribution for 10xATCT sequence in construct 2, EcoRI restriction digest control. The y-axis indicates the number of SMRT sequence reads corresponding to the sequence length (bp) indicated on the x-axis.
(BMP)

S20 Fig **Repeat length distribution for 10xATCT sequence in construct 2, 10 PCR cycles replicates 1 (A) and 2 (B).** The y-axis indicates the number of SMRT sequence reads corresponding to the sequence length (bp) indicated on the x-axis.
(BMP)

S21 Fig **Repeat length distribution for 10xATCT sequence in construct 2, 20 PCR cycles replicates 1 (A) and 2 (B).** The y-axis indicates the number of SMRT sequence reads corresponding to the sequence length (bp) indicated on the x-axis.
(BMP)

S22 Fig **Repeat length distribution for 10xATCT sequence in construct 2, 30 PCR cycles replicates 1 (A) and 2 (B).** The y-axis indicates the number of SMRT sequence reads corresponding to the sequence length (bp) indicated on the x-axis.
(BMP)

S23 Fig **Repeat length distribution for 10xATCT sequence in construct 2, 40 PCR cycles replicates 1 (A) and 2 (B).** The y-axis indicates the number of SMRT sequence reads corresponding to the sequence length (bp) indicated on the x-axis.
(BMP)

S24 Fig Absolute error density plot. Each point in the plots represents an absolute error of the magnitude (in bp) indicated on the y-axes, while the number of PCR cycles is indicated on the x-axes. Each band in the figure illustrates the density of reads deviating from the target length (zero on y-axes), with a small amount of jitter, both horizontal and vertical, added to the plotted values for enhanced visualization and interpretability. For the same reason extreme outliers have been omitted from the plots. The red lines are mean absolute percentage errors (including outliers). For each PCR regime all experimental replicates have been combined. A. 30xCA repeat sequence. B. 20xATCT repeat sequence. C. 10xATCT repeat sequence.
(BMP)

S25 Fig Relationship between PCR regime and percentage correct read length. The lines are fitted GAMs with 2 s.e. confidence regions delimited by the shaded bands. Panels A-C include the restriction digest controls while D-F do not. A. 30xCA: p = 0.058. B. 20xATCT: p<0.001. C. 10xATCT: p<0.001. D. 30xCA: p = 0.479. B. 20xATCT: p = 0.028. C. 10xATCT: p = 0.123.
(BMP)

S26 Fig Relationship between PCR regime and Shannon entropy. The lines are fitted GAMs with 2 s.e. confidence regions delimited by the shaded bands. Panels A-C include the restriction digest controls while D-F do not. A. 30xCA: p = 0.059. B. 20xATCT: p = 0.002. C. 10xATCT: p<0.001. D. 30xCA: p = 0.373. B. 20xATCT: p = 0.400. C. 10xATCT: p = 0.185
(BMP)

S27 Fig **Yield from PCR reactions using 10, 20, 30 and 40 reaction cycles for constructs 1 (A) and 2 (B).** Each reaction was done in triplicate. Error bars are ± 1 s.e.
(BMP)

S28 Fig **Relationship between stepwise deletion (A-C) and insertion (D-F) mutations and PCR cycle number.** The y-axes indicate the percentage of sequence reads corresponding to either stepwise deletions or insertions. A and D: 30xCA. B and E: 20xATCT. C and F: 10xATCT. All models (GAMs) are statistically significant. A. 30xCA: p = 0.004. B. 20xATCT: p<0.001. C. 10xATCT: p = 0.001. D. 30xCA: p = 0.005. E. 20xATCT: p = 0.024. F. 10xATCT: p = 0.006.
(BMP)

S29 Fig **Relationship beween stepwise deletion (A-C) and insertion (D-F) mutations and PCR cycle number, omitting the no-PCR controls.** The y-axes indicate the percentage of sequence reads corresponding to either stepwise deletions or insertions. A and D: 30xCA. B and E: 20xATCT. C and F: 10xATCT. Only the models (GAMs) shown in A and D are statistically significant (p = 0.027 and p = 0.024, respectively).
(BMP)

S30 Fig Percentage of errors causing reads to deviate from the target length by one nucleotide. All pairwise differences are statistically significant: 30xCA: p = 0.014, 20xATCT: p = 0.004, 10xATCT: p<0.001, linker: p<0.001 (paired Wilcoxon signed rank test). I–insertion. D–deletion.
(BMP)

S31 Fig Relationship between non-stepwise indel mutations and PCR regime. A-C: insertions. D-F: deletions. None of the relationships are statistically significant (generalized additive models). A. 30xCA: p = 0.341. B. 20xATCT: p = 0.771. C. 10xATCT: p = 0.949. D. 30xCA: p = 0.628. E. 20xATCT: p = 0.708. F. 10xATCT: p = 0.071.
(BMP)

S32 Fig Effect of PCR regime on indel rates in spacer regions. The y-axis shows the percentage of total sequence reads with an indel mutation in the spacer region of the constructs. The trend line is from a linear regression model (p = 0.006). GAM modelling also indicated a linear fit.
(BMP)

S33 Fig Mean non-stepwise indel rates normalized to sequence length. Sequence type and indel direction (I–insertions. D–deletion) are indicated on the x-axis
(BMP)

S1 File Raw sequence data. (ZIP)

S2 File R script. Utility functions for carrying out analysis.
(R) Click here for additional data file.

S3 File R script. Read and structure data.
(R) Click here for additional data file.

S4 File R script Carry out analysis. (R)

S1 Table Construct forward and reverse primers (standard desalting purification). (DOCX)

S2 Table Primer barcodes for multiplexed SMRT sequencing. All barcodes were added to 5'-end of forward primers.
(DOCX)

S3 Table Sequencing statistics contingent on number of passes used for determining CCS. (XLSX)
