# Supplementary figures and images for: Microsatellite Length Scoring by Single Molecule Real Time Sequencing – Effects of Sequence Structure and PCR Regime
Source: PLoS One. 2016 Jul 14;11(7):e0159232. doi: 10.1371/journal.pone.0159232 (PMC4945053; doi:10.1371/journal.pone.0159232)

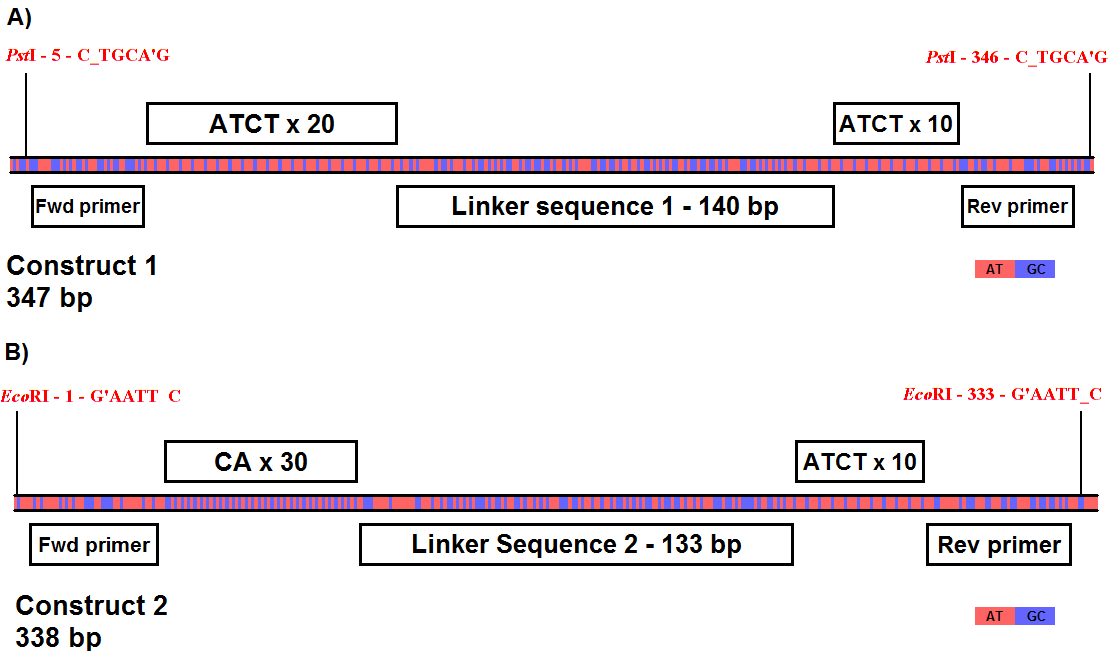

Supplement: S1 Fig — (PNG) [file pone.0159232.s001.png]

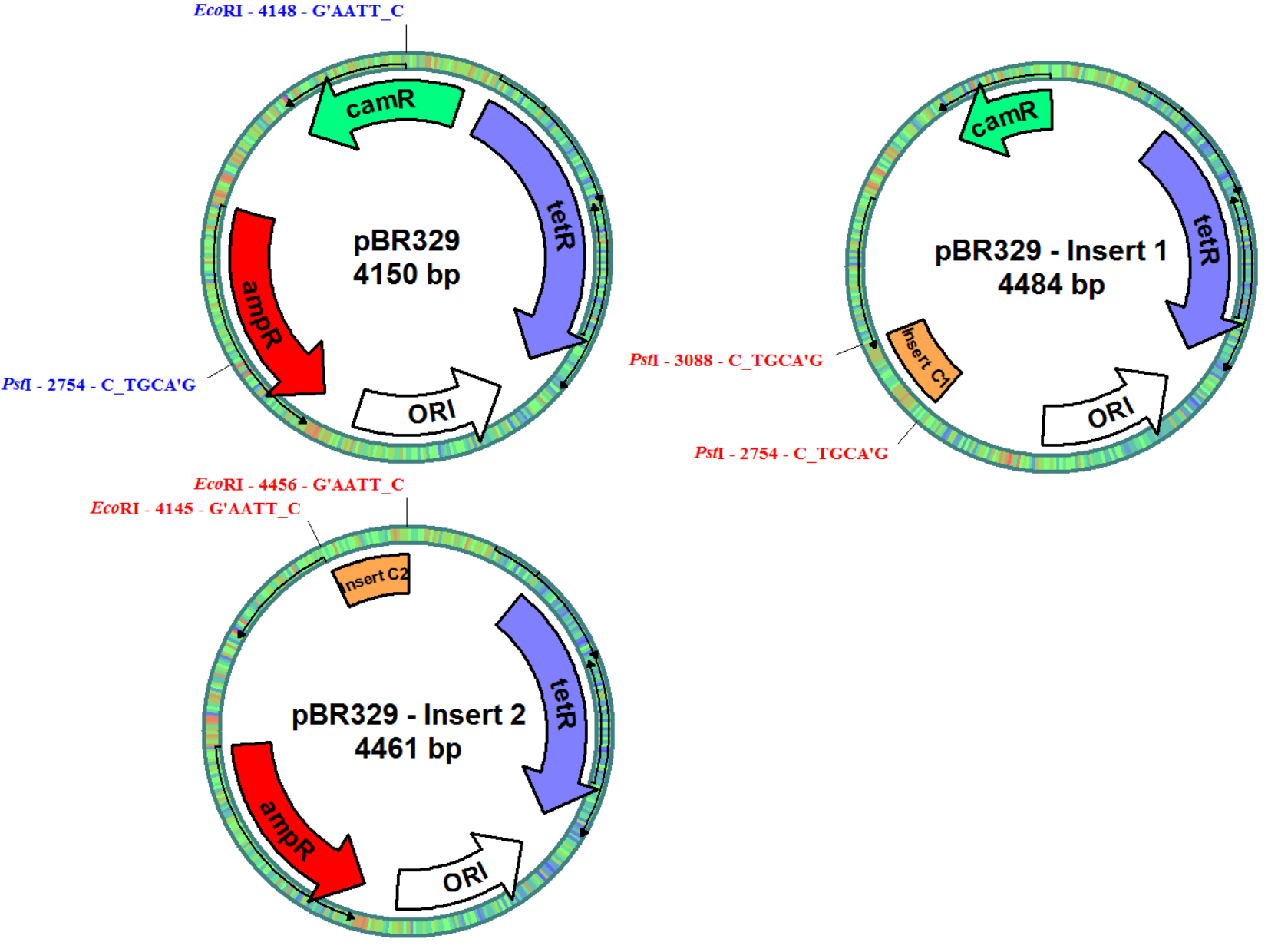

Supplement: S2 Fig — Construct 1 disrupts ampR gene, allowing for selection for ampicillin-sensitive hosts. Construct 2 disrupts camR gene, allowing for selection for chlorampenicol-sensitive hosts. Both vectors provide tetracycline resistance to host. (PNG) [file pone.0159232.s002.png]

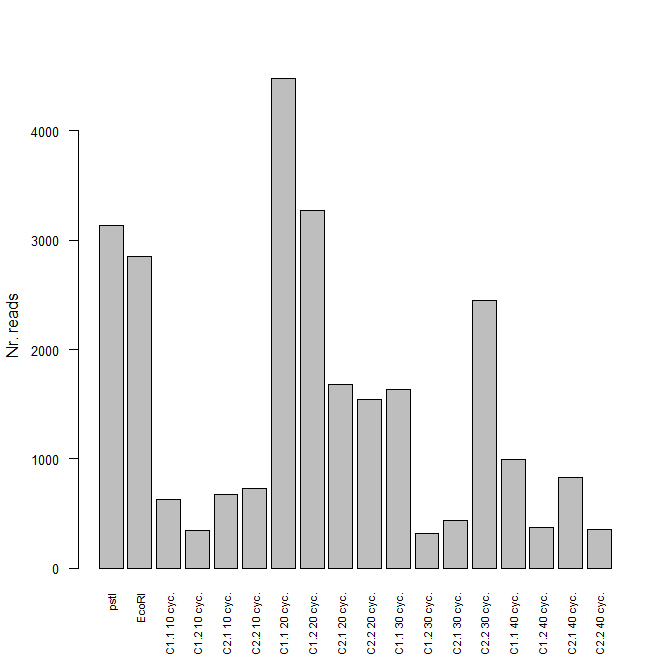

Supplement: S3 Fig — The total number of reads for each experimental treatment in indicated on the y-axis. Experimental treatment is indicated is indicated on the x-axis. EcoRI and pstI are the no-PCR controls. Construct number (C1 or C2) is indicated first in the other x-axis labels, followed by replicate number (1 or 2), followed by number of PCR cycles (10–40). (BMP) [file pone.0159232.s003.bmp]

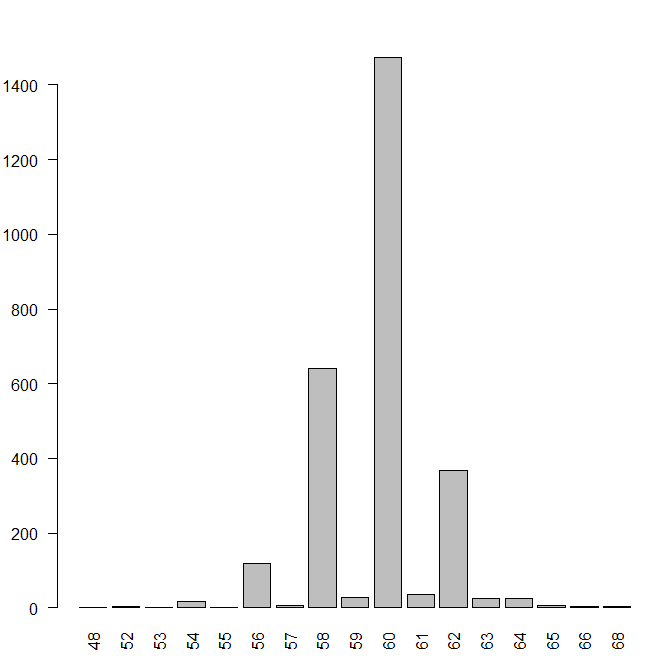

Supplement: S4 Fig — The y-axis indicates the number of SMRT sequence reads corresponding to the sequence length (bp) indicated on the x-axis. (BMP) [file pone.0159232.s004.bmp]

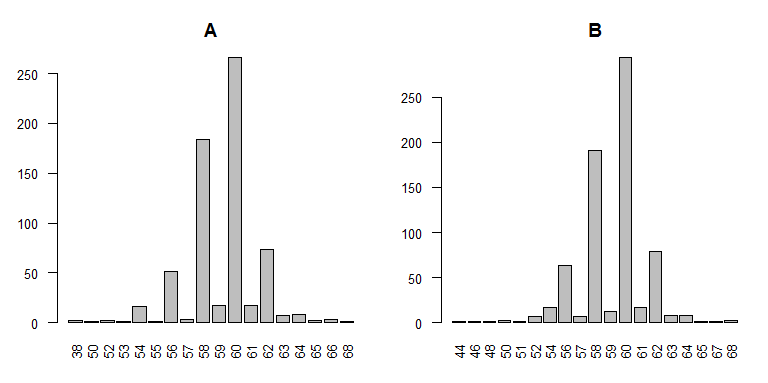

Supplement: S5 Fig — Repeat length distribution for 30xCA sequence, 10 PCR cycles replicates 1 (A) and 2 (B). The y-axis indicates the number of SMRT sequence reads corresponding to the sequence length (bp) indicated on the x-axis. (BMP) [file pone.0159232.s005.bmp]

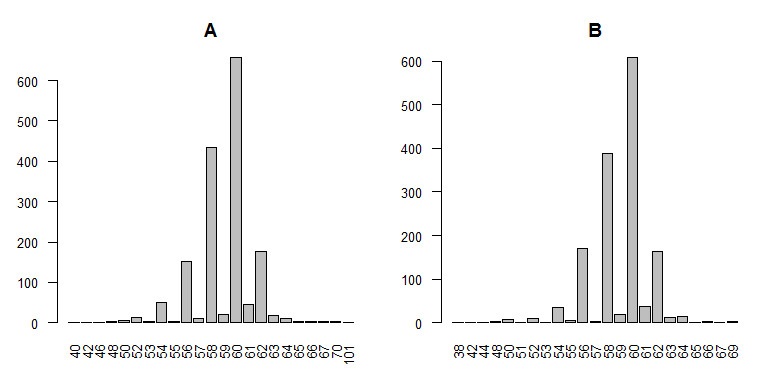

Supplement: S6 Fig — Repeat length distribution for 30xCA sequence, 20 PCR cycles replicates 1 (A) and 2 (B). The y-axis indicates the number of SMRT sequence reads corresponding to the sequence length (bp) indicated on the x-axis. (BMP) [file pone.0159232.s006.bmp]

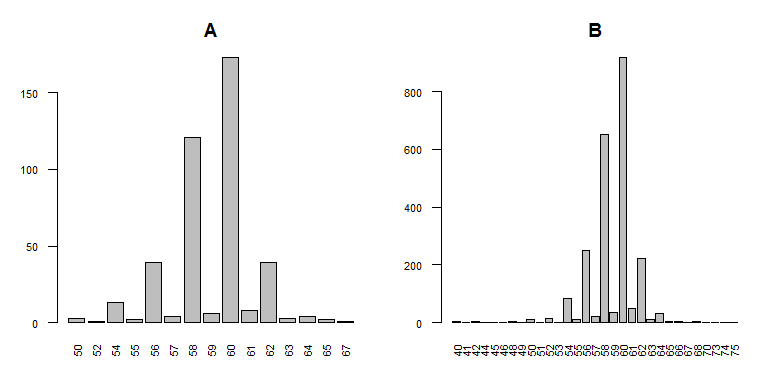

Supplement: S7 Fig — Repeat length distribution for 30xCA sequence, 30 PCR cycles replicates 1 (A) and 2 (B). The y-axis indicates the number of SMRT sequence reads corresponding to the sequence length (bp) indicated on the x-axis. (BMP) [file pone.0159232.s007.bmp]

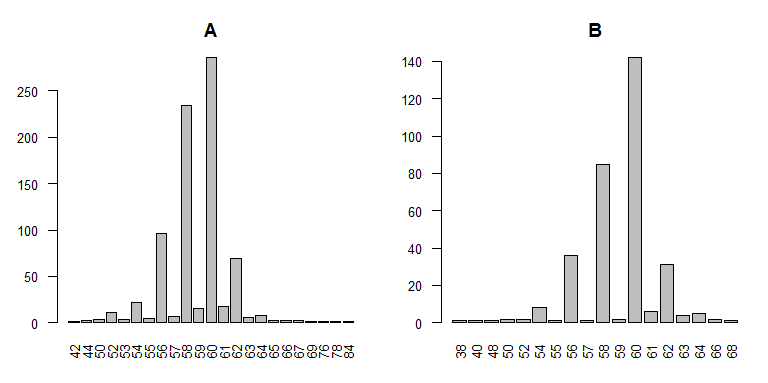

Supplement: S8 Fig — Repeat length distribution for 30xCA sequence, 40 PCR cycles replicates 1 (A) and 2 (B). The y-axis indicates the number of SMRT sequence reads corresponding to the sequence length (bp) indicated on the x-axis. (BMP) [file pone.0159232.s008.bmp]

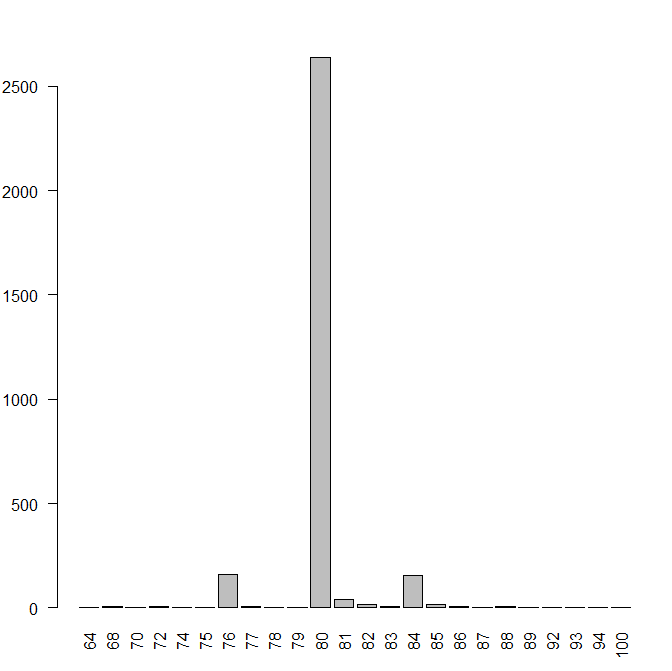

Supplement: S9 Fig — The y-axis indicates the number of SMRT sequence reads corresponding to the sequence length (bp) indicated on the x-axis. (BMP) [file pone.0159232.s009.bmp]

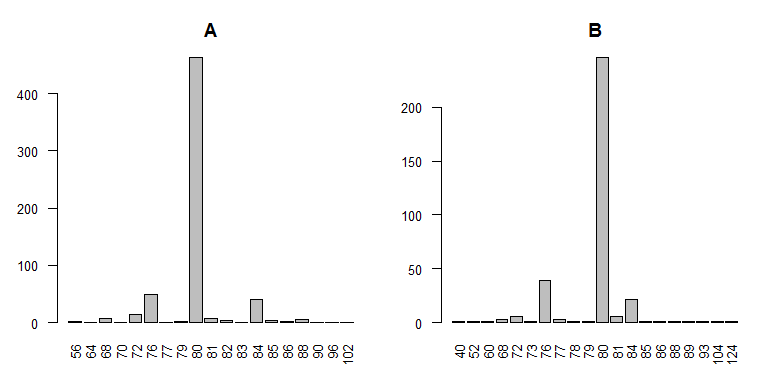

Supplement: S10 Fig — Repeat length distribution for 20xATCT sequence, 10 PCR cycles replicates 1 (A) and 2 (B). The y-axis indicates the number of SMRT sequence reads corresponding to the sequence length (bp) indicated on the x-axis. (BMP) [file pone.0159232.s010.bmp]

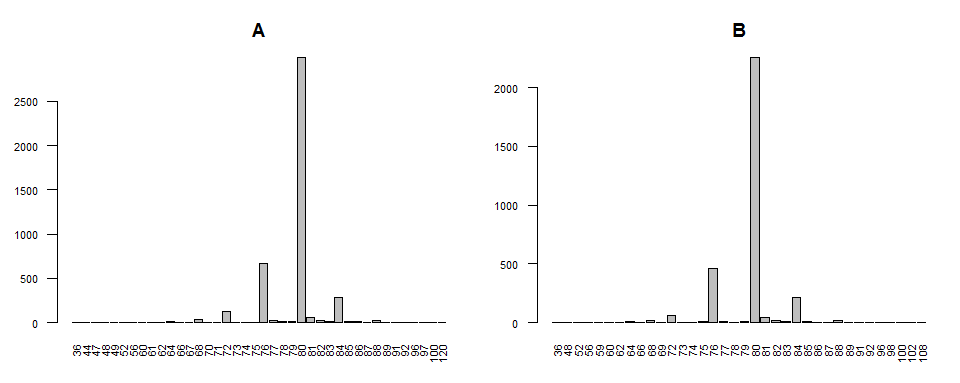

Supplement: S11 Fig — Repeat length distribution for 20xATCT sequence, 20 PCR cycles replicates 1 (A) and 2 (B). The y-axis indicates the number of SMRT sequence reads corresponding to the sequence length (bp) indicated on the x-axis. (BMP) [file pone.0159232.s011.bmp]

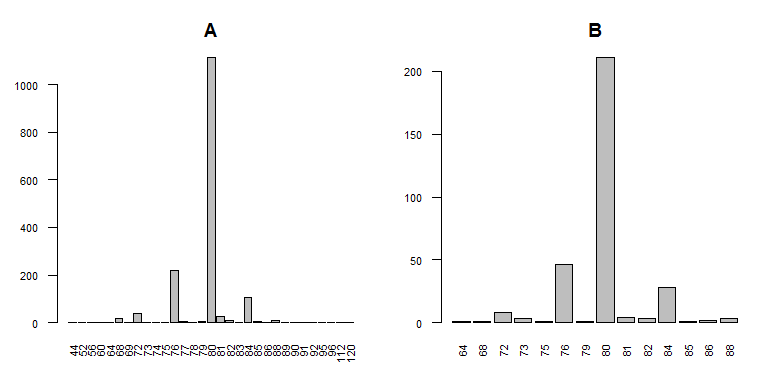

Supplement: S12 Fig — Repeat length distribution for 20xATCT sequence, 30 PCR cycles replicates 1 (A) and 2 (B). The y-axis indicates the number of SMRT sequence reads corresponding to the sequence length (bp) indicated on the x-axis. (BMP) [file pone.0159232.s012.bmp]

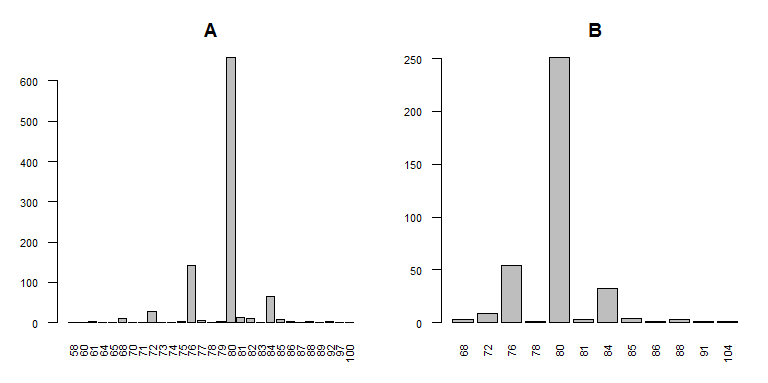

Supplement: S13 Fig — Repeat length distribution for 20xATCT sequence, 40 PCR cycles replicates 1 (A) and 2 (B). The y-axis indicates the number of SMRT sequence reads corresponding to the sequence length (bp) indicated on the x-axis. (BMP) [file pone.0159232.s013.bmp]

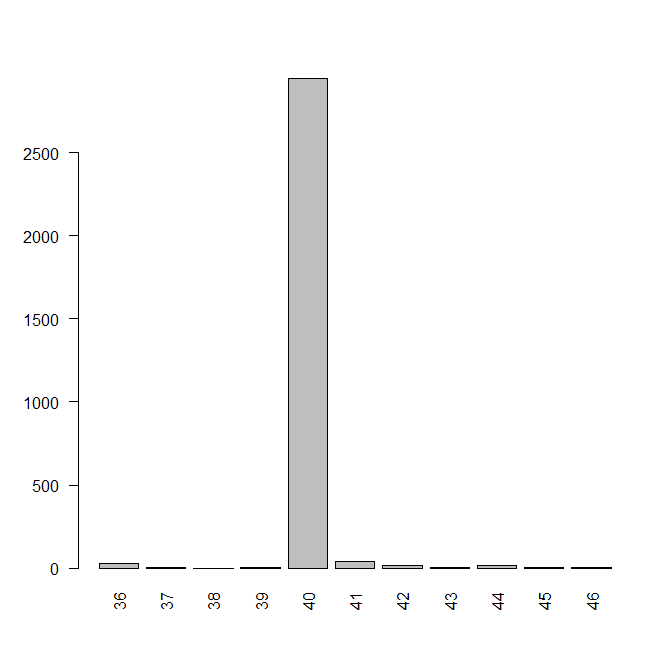

Supplement: S14 Fig — The y-axis indicates the number of SMRT sequence reads corresponding to the sequence length (bp) indicated on the x-axis. (BMP) [file pone.0159232.s014.bmp]

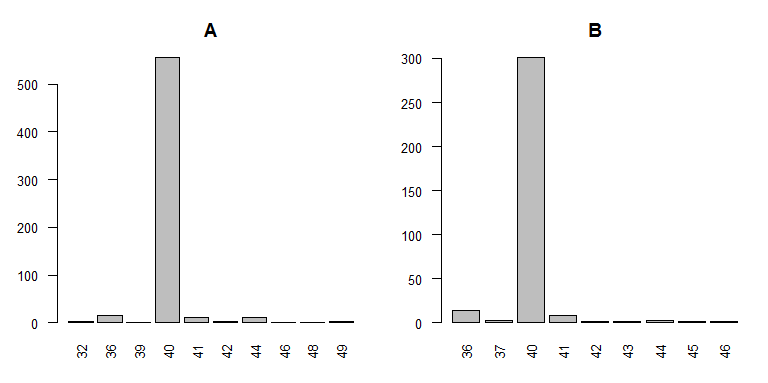

Supplement: S15 Fig — Repeat length distribution for 10xATCT sequence in construct 1, 10 PCR cycles replicates 1 (A) and 2 (B). The y-axis indicates the number of SMRT sequence reads corresponding to the sequence length (bp) indicated on the x-axis. (BMP) [file pone.0159232.s015.bmp]

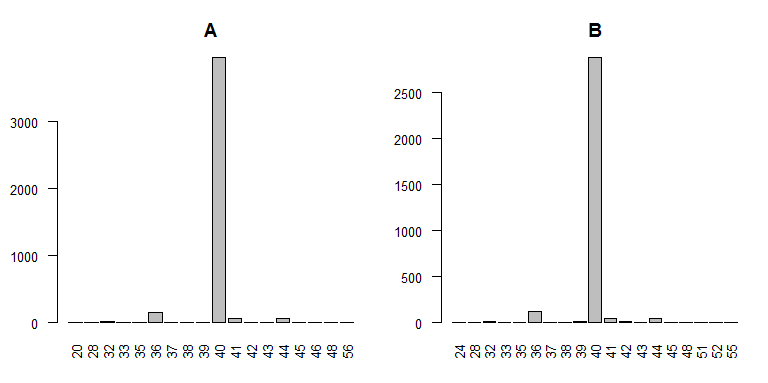

Supplement: S16 Fig — Repeat length distribution for 10xATCT sequence in construct 1, 20 PCR cycles replicates 1 (A) and 2 (B). The y-axis indicates the number of SMRT sequence reads corresponding to the sequence length (bp) indicated on the x-axis. (BMP) [file pone.0159232.s016.bmp]

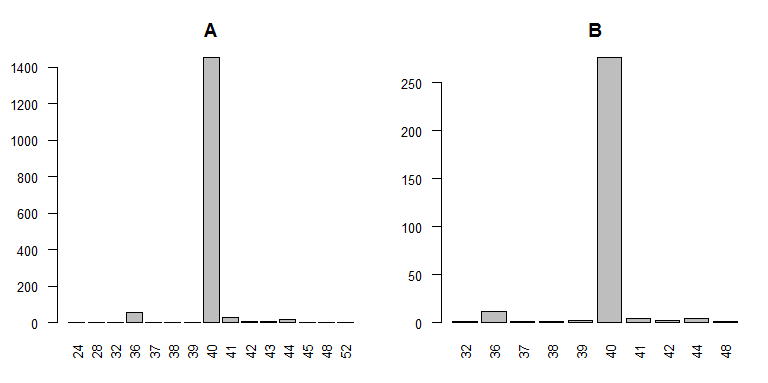

Supplement: S17 Fig — Repeat length distribution for 10xATCT sequence in construct 1, 30 PCR cycles replicates 1 (A) and 2 (B). The y-axis indicates the number of SMRT sequence reads corresponding to the sequence length (bp) indicated on the x-axis. (BMP) [file pone.0159232.s017.bmp]

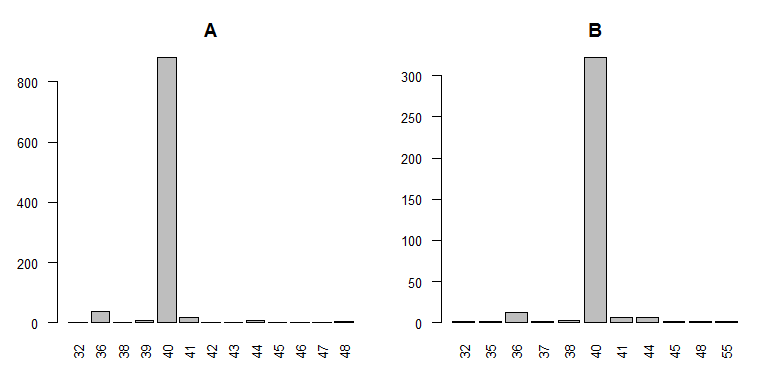

Supplement: S18 Fig — Repeat length distribution for 10xATCT sequence in construct 1, 40 PCR cycles replicates 1 (A) and 2 (B). The y-axis indicates the number of SMRT sequence reads corresponding to the sequence length (bp) indicated on the x-axis. (BMP) [file pone.0159232.s018.bmp]

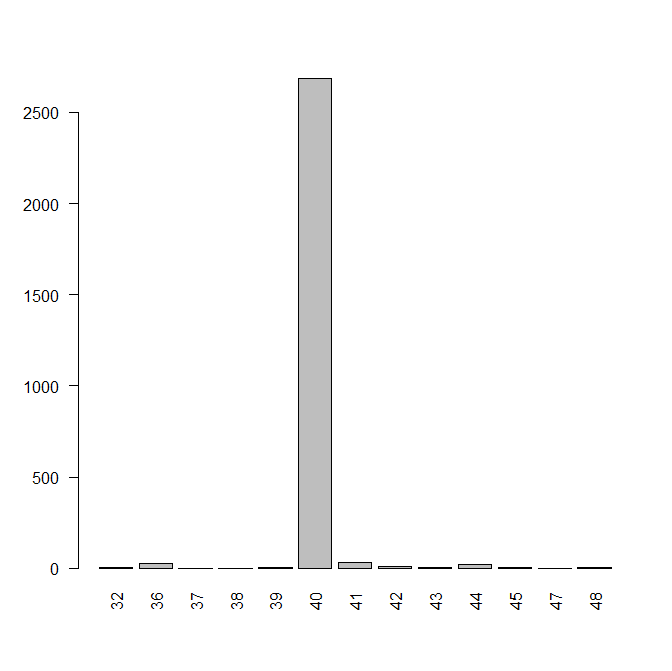

Supplement: S19 Fig — The y-axis indicates the number of SMRT sequence reads corresponding to the sequence length (bp) indicated on the x-axis. (BMP) [file pone.0159232.s019.bmp]

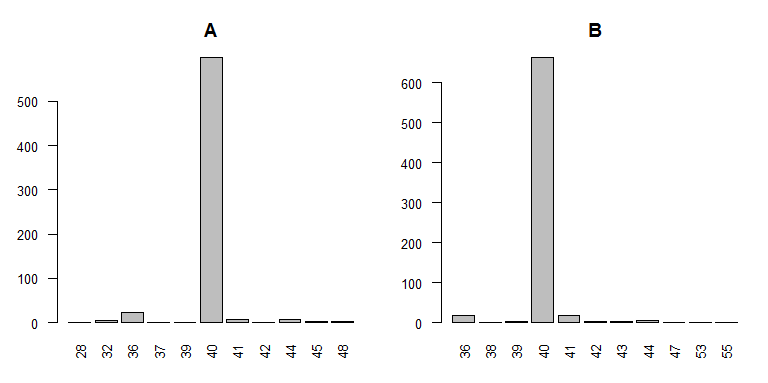

Supplement: S20 Fig — Repeat length distribution for 10xATCT sequence in construct 2, 10 PCR cycles replicates 1 (A) and 2 (B). The y-axis indicates the number of SMRT sequence reads corresponding to the sequence length (bp) indicated on the x-axis. (BMP) [file pone.0159232.s020.bmp]

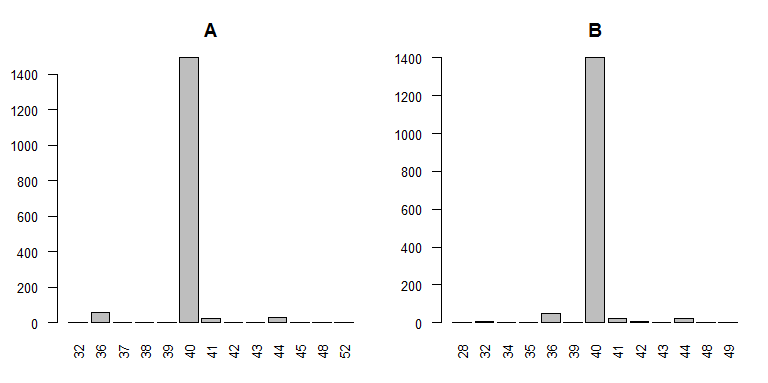

Supplement: S21 Fig — Repeat length distribution for 10xATCT sequence in construct 2, 20 PCR cycles replicates 1 (A) and 2 (B). The y-axis indicates the number of SMRT sequence reads corresponding to the sequence length (bp) indicated on the x-axis. (BMP) [file pone.0159232.s021.bmp]

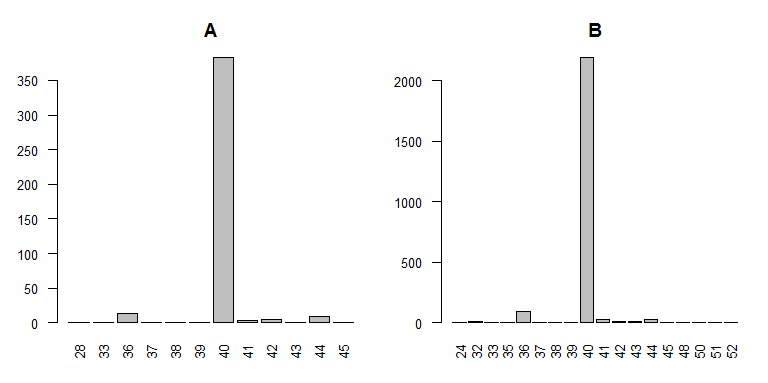

Supplement: S22 Fig — Repeat length distribution for 10xATCT sequence in construct 2, 30 PCR cycles replicates 1 (A) and 2 (B). The y-axis indicates the number of SMRT sequence reads corresponding to the sequence length (bp) indicated on the x-axis. (BMP) [file pone.0159232.s022.bmp]

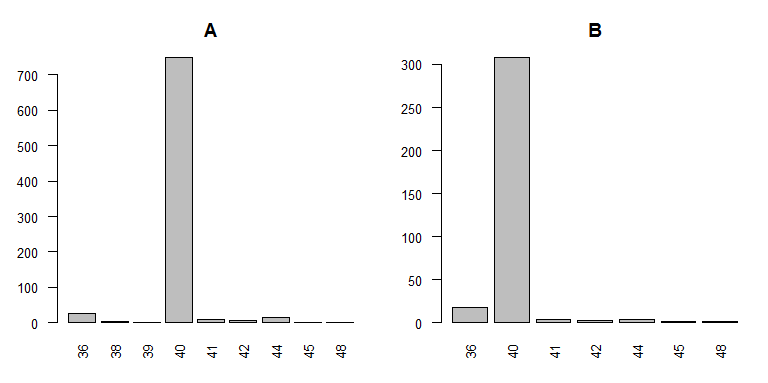

Supplement: S23 Fig — Repeat length distribution for 10xATCT sequence in construct 2, 40 PCR cycles replicates 1 (A) and 2 (B). The y-axis indicates the number of SMRT sequence reads corresponding to the sequence length (bp) indicated on the x-axis. (BMP) [file pone.0159232.s023.bmp]

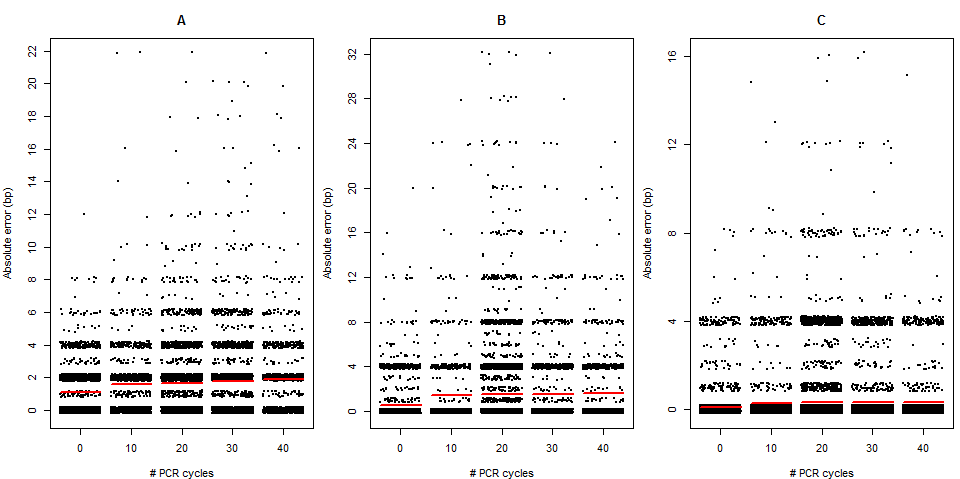

Supplement: S24 Fig — Each point in the plots represents an absolute error of the magnitude (in bp) indicated on the y-axes, while the number of PCR cycles is indicated on the x-axes. Each band in the figure illustrates the density of reads deviating from the target length (zero on y-axes), with a small amount of jitter, both horizontal and vertical, added to the plotted values for enhanced visualization and interpretability. For the same reason extreme outliers have been omitted from the plots. The red lines are mean absolute percentage errors (including outliers). For each PCR regime all experimental replicates have been combined. A. 30xCA repeat sequence. B. 20xATCT repeat sequence. C. 10xATCT repeat sequence. (BMP) [file pone.0159232.s024.bmp]

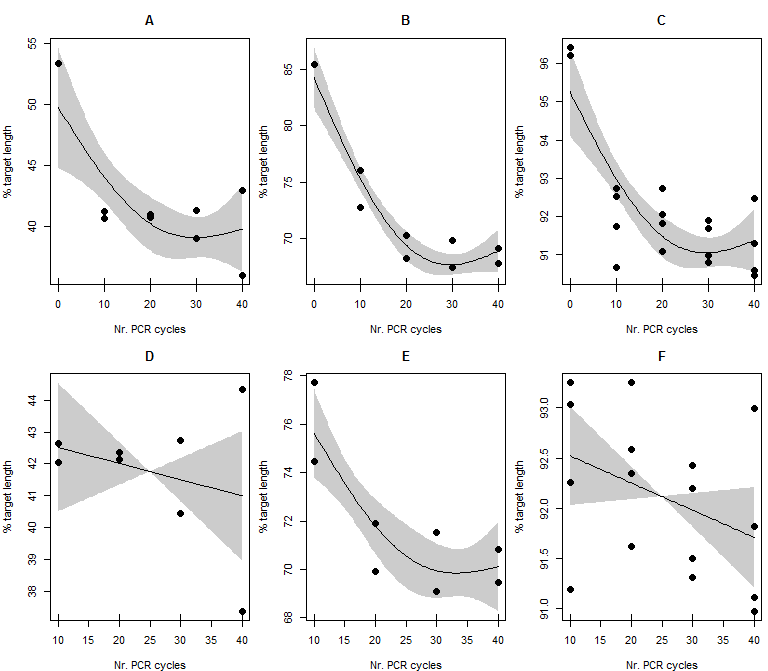

Supplement: S25 Fig — The lines are fitted GAMs with 2 s.e. confidence regions delimited by the shaded bands. Panels A-C include the restriction digest controls while D-F do not. A. 30xCA: p = 0.058. B. 20xATCT: p<0.001. C. 10xATCT: p<0.001. D. 30xCA: p = 0.479. B. 20xATCT: p = 0.028. C. 10xATCT: p = 0.123. (BMP) [file pone.0159232.s025.bmp]

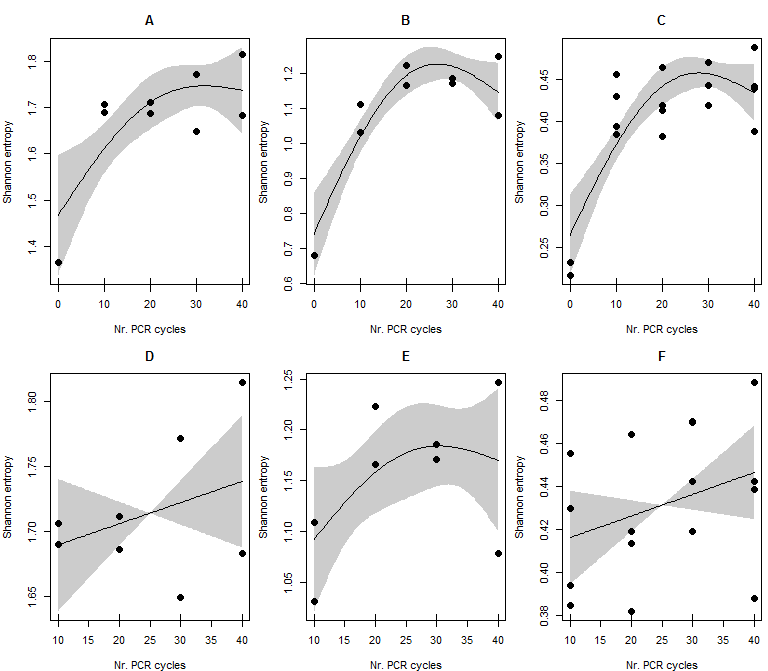

Supplement: S26 Fig — The lines are fitted GAMs with 2 s.e. confidence regions delimited by the shaded bands. Panels A-C include the restriction digest controls while D-F do not. A. 30xCA: p = 0.059. B. 20xATCT: p = 0.002. C. 10xATCT: p<0.001. D. 30xCA: p = 0.373. B. 20xATCT: p = 0.400. C. 10xATCT: p = 0.185 (BMP) [file pone.0159232.s026.bmp]

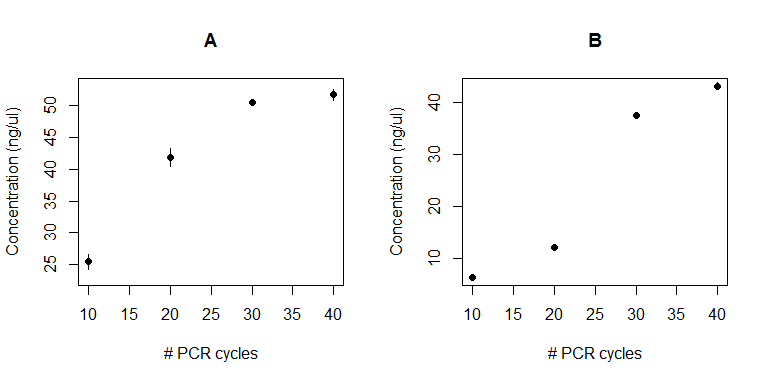

Supplement: S27 Fig — Yield from PCR reactions using 10, 20, 30 and 40 reaction cycles for constructs 1 (A) and 2 (B). Each reaction was done in triplicate. Error bars are ± 1 s.e. (BMP) [file pone.0159232.s027.bmp]

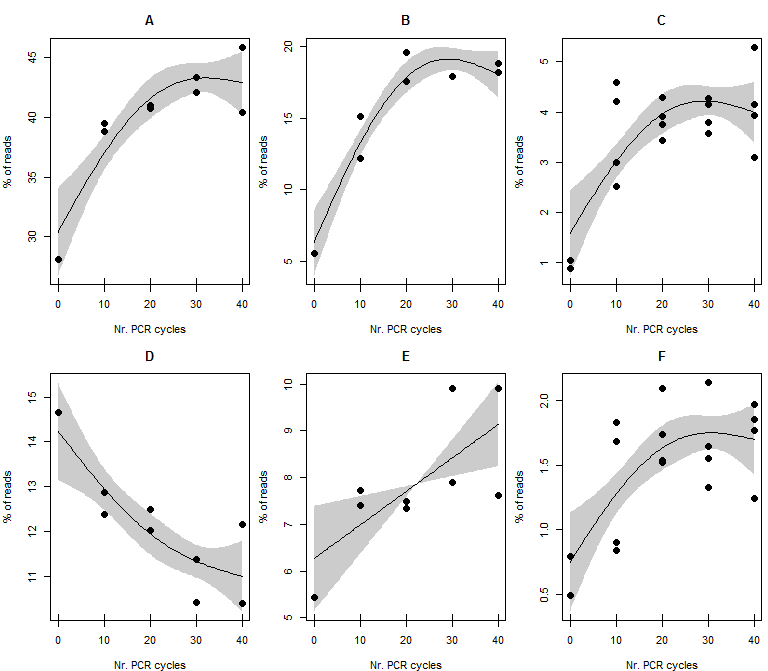

Supplement: S28 Fig — Relationship between stepwise deletion (A-C) and insertion (D-F) mutations and PCR cycle number. The y-axes indicate the percentage of sequence reads corresponding to either stepwise deletions or insertions. A and D: 30xCA. B and E: 20xATCT. C and F: 10xATCT. All models (GAMs) are statistically significant. A. 30xCA: p = 0.004. B. 20xATCT: p<0.001. C. 10xATCT: p = 0.001. D. 30xCA: p = 0.005. E. 20xATCT: p = 0.024. F. 10xATCT: p = 0.006. (BMP) [file pone.0159232.s028.bmp]

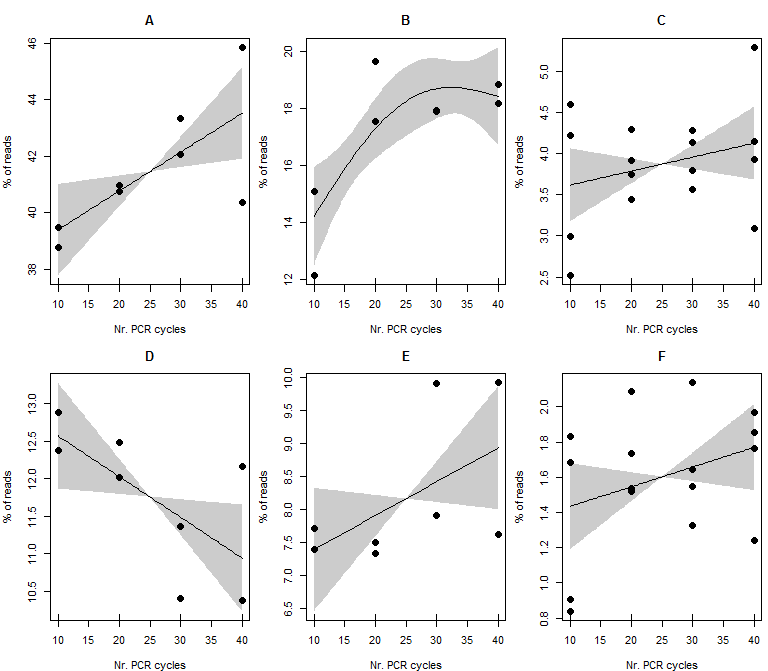

Supplement: S29 Fig — Relationship beween stepwise deletion (A-C) and insertion (D-F) mutations and PCR cycle number, omitting the no-PCR controls. The y-axes indicate the percentage of sequence reads corresponding to either stepwise deletions or insertions. A and D: 30xCA. B and E: 20xATCT. C and F: 10xATCT. Only the models (GAMs) shown in A and D are statistically significant (p = 0.027 and p = 0.024, respectively). (BMP) [file pone.0159232.s029.bmp]

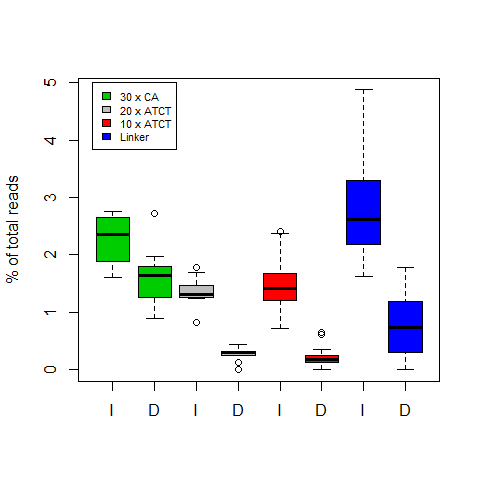

Supplement: S30 Fig — All pairwise differences are statistically significant: 30xCA: p = 0.014, 20xATCT: p = 0.004, 10xATCT: p<0.001, linker: p<0.001 (paired Wilcoxon signed rank test). I–insertion. D–deletion. (BMP) [file pone.0159232.s030.bmp]

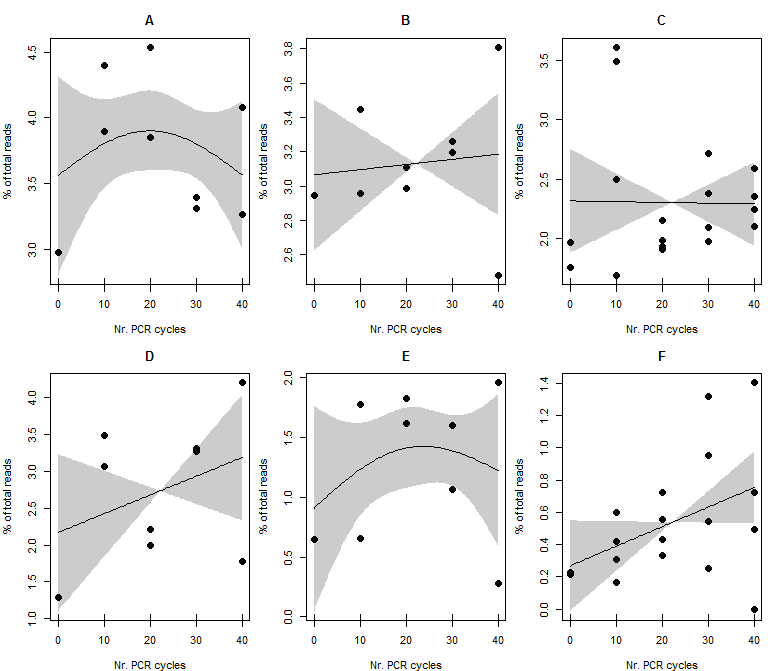

Supplement: S31 Fig — A-C: insertions. D-F: deletions. None of the relationships are statistically significant (generalized additive models). A. 30xCA: p = 0.341. B. 20xATCT: p = 0.771. C. 10xATCT: p = 0.949. D. 30xCA: p = 0.628. E. 20xATCT: p = 0.708. F. 10xATCT: p = 0.071. (BMP) [file pone.0159232.s031.bmp]

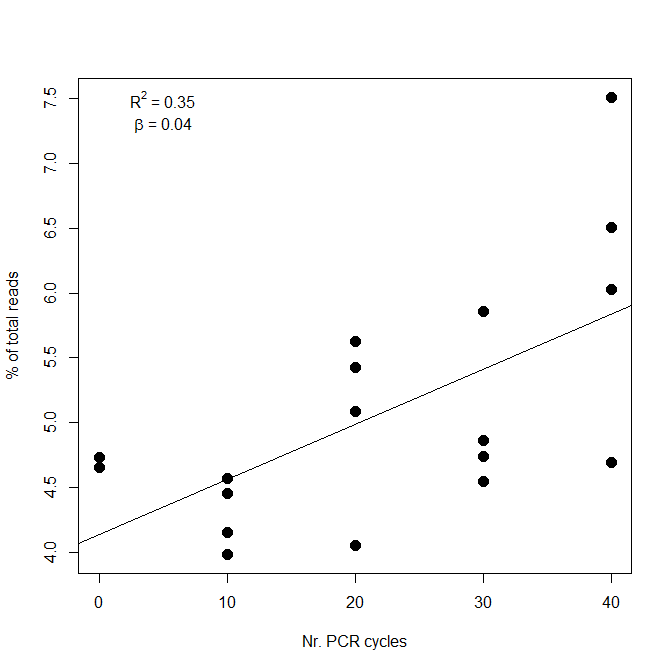

Supplement: S32 Fig — The y-axis shows the percentage of total sequence reads with an indel mutation in the spacer region of the constructs. The trend line is from a linear regression model (p = 0.006). GAM modelling also indicated a linear fit. (BMP) [file pone.0159232.s032.bmp]

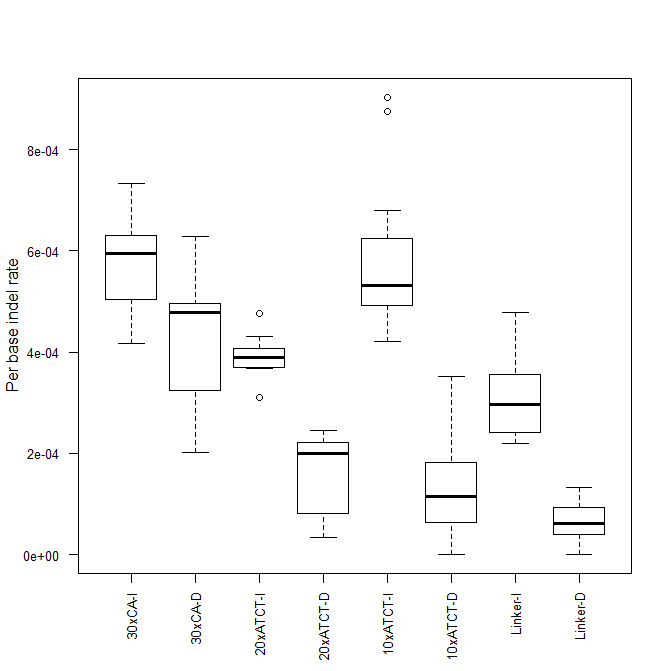

Supplement: S33 Fig — Sequence type and indel direction (I–insertions. D–deletion) are indicated on the x-axis (BMP) [file pone.0159232.s033.bmp]
