# Supplementary material for: Microsatellite Length Scoring by Single Molecule Real Time Sequencing – Effects of Sequence Structure and PCR Regime
Source: PLoS One. 2016 Jul 14;11(7):e0159232. doi: 10.1371/journal.pone.0159232 (PMC4945053; doi:10.1371/journal.pone.0159232)
Supplement: S1 Table — (DOCX) [file pone.0159232.s038.docx]

| **Construct number:** | **Forward primer (5'):** | **Reverse primer (3'):** |
| --- | --- | --- |
| 1 | CTGCAGGGTTTTCCCAGTC | CTGCAGTCACACAGGAAACA |
| 2 | GAATTCACCGTTGTAACCAA | GAATTCGTTTGACACCTGCT |
