# Supplementary material for: Microsatellite Length Scoring by Single Molecule Real Time Sequencing – Effects of Sequence Structure and PCR Regime
Source: PLoS One. 2016 Jul 14;11(7):e0159232. doi: 10.1371/journal.pone.0159232 (PMC4945053; doi:10.1371/journal.pone.0159232)
Supplement: S2 Table — All barcodes were added to 5'-end of forward primers. (DOCX) [file pone.0159232.s039.docx]

| **Barcode #:** | **Sequence:** |
| --- | --- |
| Construct 1 replicate 1, 10 cycles | AACCCCTG |
| Construct 2 replicate 1, 10 cycles | AACCCGAA |
| Construct 1 replicate 2, 10 cycles | ACACGCTG |
| Construct 2 replicate 2, 10 cycles | ACACGGAA |
| Construct 1 replicate 1, 20 cycles | ACTGCCTG |
| Construct 2 replicate 1, 20 cycles | ACTGCGAA |
| Construct 1 replicate 2, 20 cycles | AGAGTCTG |
| Construct 2 replicate 2, 20 cycles | AGAGTGAA |
| Construct 1 replicate 1, 30 cycles | AGCTGCTG |
| Construct 2 replicate 1, 30 cycles | AGCTGGAA |
| Construct 1 replicate 2, 30 cycles | AGGACCTG |
| Construct 2 replicate 2, 30 cycles | AGGACGAA |
| Construct 1 replicate 1, 40 cycles | AGTCACTG |
| Construct 2 replicate 1, 40 cycles | AGTCAGAA |
| Construct 1 replicate 2, 40 cycles | ATATCCTG |
| Construct 2 replicate 2, 40 cycles | ATATCGAA |
